# Word frequency and contextual diversity measures for Singapore English

**DOI:** 10.3758/s13428-026-03012-1

**Published:** 2026-04-29

**Authors:** Zehua R. Jiang, Cynthia S. Q. Siew

**Affiliations:** 1 Department of Psychology, National University of Singapore, 9 Arts Link, Block AS4, Singapore, 117570 Singapore; 2 Department of Psychology and Behavioral Sciences, Zhejiang University, Hangzhou, China

**Keywords:** Singapore English, Word frequency, Contextual diversity, English dialects

## Abstract

Singapore English, shaped by unique historical and sociocultural factors, diverges significantly from American and British English varieties. Despite its centrality in Singapore’s communication landscape, comprehensive word frequency estimates for this dialect have been lacking, limiting psycholinguistic research that accurately reflects local language processing. Dialectal variation fundamentally influences cognitive mechanisms, and neglecting these differences can distort theoretical models of language processing. This study addresses this gap by constructing word frequency (WF) and contextual diversity (CD) estimates from the National Speech Corpus (NSC), a large-scale speech corpus of Singapore English. We analyzed three NSC subparts comprising over 10,000 transcripts and 30 + million words to generate frequency estimates for 50,000 words, including both standard and uniquely Singaporean terms. To validate these measures, we used lexical decision data from the Auditory English Lexicon Project, featuring Singaporean participants who responded to stimuli across different accent conditions. Linear and binomial mixed-effect models compared NSC-based estimates against SUBTLEX-US and SUBTLEX-UK in predicting reaction times and accuracy. NSC estimates produced the lowest Akaike information criterion (AIC) values across both British and Singaporean accents, indicating superior fit to local language processing patterns. These results demonstrate that WF and CD effects are shaped by specific language experiences and highlight the necessity of dialect-appropriate psycholinguistic tools. This database advances cognitive science by enabling more accurate modeling of dialect-sensitive language processing, representing one of the largest psycholinguistic resources available for a less-studied English variety, and underscores the importance of developing similar resources for other English varieties worldwide.

**Supplementary Information:**

The online version contains supplementary material available at 10.3758/s13428-026-03012-1.

## Introduction

Word frequency effects are fundamental to understanding word recognition processes. Extensive studies have demonstrated that word frequency, which reflects individuals’ language exposure, consistently influences various aspects of human cognition (Brysbaert et al., 2018). Given its critical role in cognitive processing, researchers have developed word frequency databases for many languages to quantify speakers’ linguistic experience (Boada et al., 2020; Boudelaa et al., 2025; Brysbaert & New, 2009; Brysbaert et al., 2012; Cai & Brysbaert, 2010; Duchon et al., 2013; Keuleers et al., 2010a; Mandera et al., 2015; van Heuven et al., 2014, 2024).

Word frequency databases for English primarily capture word usage patterns in dominant varieties of English, such as North American English and British English. This is exemplified by two highly popular word frequency databases based on movie and television subtitles, SUBTLEX-US (Brysbaert et al., 2012) and SUBTLEX-UK (van Heuven et al., 2014), representing American and British English, respectively. However, this focus on mainstream varieties creates a significant methodological problem in psycholinguistic research. When researchers use mainstream varieties as proxies for all other varieties, they implicitly assume uniform linguistic experiences across participants, thereby overlooking crucial differences in how different populations actually use language (Henrich et al., 2010; Levisen, 2019). Hence, the development of measures that capture the linguistic experiences of diverse populations enables psycholinguists to better investigate both the similarities and differences in cognitive mechanisms across languages and dialects. This methodological concern becomes particularly significant when we recognize that “English” encompasses a diverse group of varieties.

Within English itself, numerous dialects shaped by distinct historical and cultural factors diverge significantly from mainstream varieties, raising important questions about the generalizability of findings across English varieties (Kutlu & Hayes-Harb, 2023; Levisen, 2019). The overrepresentation of so-called standard English in research compromises both scientific validity and practical applications. For instance, Brown et al. (2023) attempted to replicate Reid et al. (2019)—which originally found that native English speakers exhibited socially oriented bias toward non-native English speakers—with participants across different countries who were native English speakers according to traditional psycholinguistic definitions. They found no evidence of the effect reported in Reid et al.’s original study, highlighting how dialectal diversity can influence research outcomes.

Furthermore, the significance of intra-linguistic variation extends to fundamental cognitive processing mechanisms and development (Byrd et al., 2023; De Villiers & Johnson, 2007; Garcia et al., 2022; Kirk, 2023; Oetting et al., 2023; Terry et al., 2015). In some cases, speakers who identify as “monolingual” process different varieties of their language in distinct ways. Scots speakers, for example, demonstrate bilingual-like characteristics when switching between standard English and their dialect, processing these varieties through separate cognitive systems despite self-identifying as monolingual English speakers (Kirk, 2023). Similarly, processing mechanisms for English can vary significantly across different populations. Byrd et al. (2023) showed that African American English-speaking children apply grammatical rules specific to their dialect when processing ambiguous sentences, rather than following mainstream English conventions. These findings demonstrate that language is fundamentally shaped by cultural context and environment and is not universally identical. Studying dialectal variations such as Singapore English is therefore essential for developing accurate models of language processing that reflect the true diversity of English speakers worldwide and avoid the limitations imposed by an overreliance on mainstream varieties.

Singapore English is the variety of English spoken in Singapore, which has been shaped by historical and sociocultural circumstances unique to Singapore, a city-state in Southeast Asia (Leimgruber, 2011). While researchers have investigated various psycholinguistic effects among participants who are native speakers of Singapore English (e.g. Goh et al., 2016, 2020; Yap et al., 2015), a comprehensive and standardized word frequency database for Singapore English remains notably absent. In such studies, researchers typically rely on word frequency statistics developed on dominant varieties of English (such as SUBTLEX-US or SUBTLEX-UK) for stimuli selection and stimulus control. This gap represents a critical limitation for psycholinguistic research in the Singaporean context, given that Siew (2024) demonstrated that Singapore-specific word norms were more closely aligned with word usage and frequency patterns in a Singaporean context as compared to a North American context. Specifically, word humor ratings are negatively associated with word frequency (i.e., funny words tend to be less frequently occurring in the language), and this relationship was significantly stronger when the norms and frequency measures were obtained from the *same* population.

Singapore English exists in a unique diglossic context encompassing both Singapore Standard English (SSE) and Singapore Colloquial English (SCE; Alsagoff, 2010). SSE, influenced by Singapore’s British colonial history, adheres to British English conventions in spelling, grammar, and pronunciation, and is predominantly used in formal contexts. In contrast, SCE, commonly known as “Singlish,” is used in informal settings and exhibits distinctive features stemming from Singapore’s multicultural society. These include unique discourse particles (e.g., “lah,” “lor”), lexical items (e.g., “kena,” “shiok”), and grammatical structures (e.g., topic prominence and reduplication) influenced by Chinese, Malay, and other local languages (Gonzales et al., 2023; Leimgruber, 2011; Wong, 2004). For instance, the increased usage of “already” in sentential constructions reflects the influence of linguistic blending between Chinese and English (Bao et al., 1998). This distinctive linguistic characteristic suggests that Singaporean English speakers may employ cognitive processing mechanisms that differ fundamentally from those of speakers of other English varieties (Brown et al., 2023; Byrd et al., 2023; Kirk, 2023). Existing word frequency databases fail to capture these unique linguistic patterns, either missing Singapore-specific terms entirely or misrepresenting the frequency of words that exist in both standard English and Singapore English but serve different functions. These limitations potentially compromise the validity of psycholinguistic research on Singapore English speakers, as standard English databases fail to reflect the actual linguistic environment experienced by Singaporeans. To accurately investigate the cognitive mechanisms underlying language processing in Singapore English speakers, researchers require tools specifically calibrated to represent the linguistic input these speakers actually encounter in their daily lives.

To address this gap, we utilized the National Speech Corpus (NSC, Koh et al., 2019), developed by Singapore’s Infocomm Media Development Authority (IMDA), to construct word frequency norms for Singapore English. The NSC is a speech corpus originally designed to build automatic speech recognition systems for Singaporean-accented English, comprising audio recordings of Singaporean speakers and their corresponding transcripts. While popular psycholinguistic word frequency databases such as SUBTLEX-US (Brysbaert et al., 2012) and SUBTLEX-UK (van Heuven et al., 2014) were derived from subtitle corpora, our use of a speech corpus was motivated by two key factors.

First, there is limited availability of Singapore English corpora of sufficient size—at least 16 million tokens are needed to build reliable word frequency estimates (Brysbaert & New, 2009). Many available resources are blended with English from other sources, whereas the NSC meets the size requirement with over 30 million tokens, a substantial corpus for a less-studied variety, and contains language produced exclusively by Singaporean speakers. Second, and more importantly, Singapore English exhibits its most distinctive features in spoken form, particularly SCE. A speech corpus is therefore especially appropriate for capturing its unique characteristics as compared to written corpora, as it better represents the daily communication patterns and linguistic experiences of Singaporean speakers. Consequently, we utilized the NSC to develop comprehensive word frequency norms for Singapore English.

To validate our frequency norms, we employed the Auditory English Lexicon Project (AELP; Goh et al., 2020), a multi-talker database containing spoken words and nonwords in Singaporean, American, and British accents. Although many other megastudies exist, it is important to note that the behavioral task was ultimately completed by individuals who do not speak Singapore English. For instance, the data in the English Lexicon Project (Balota et al., 2007) were obtained from undergraduates in a North American institution, and data in the British Lexicon Project (Keuleers et al., 2012) were obtained from undergraduates in a British institution. Since Singapore English exhibits its most distinctive features primarily in spoken form, the auditory lexical decision task better captures Singapore English linguistic representations than visual tasks. As the AELP includes auditory lexical decision task data obtained from participants who are native speakers of Singapore English in the spoken modality, it is a particularly suitable dataset for validating how well our frequency norms capture Singaporean speakers’ language exposure relative to other frequency datasets.

### The current study

The present study addresses a notable gap in psycholinguistic resources by developing comprehensive word frequency norms for Singapore English using the NSC. Our aims here are (a) to create word frequency metrics that accurately represent Singaporean English usage patterns, including its unique lexical and grammatical features, and (b) to validate these norms via an analysis of data from the AELP (Goh et al., 2020), specifically examining their predictive power across different English accents. By establishing these norms, we aim to provide researchers with lexical measures that more precisely capture the linguistic experience of Singaporean English speakers, facilitating psycholinguistic research in this unique linguistic context.

## Method

### Corpus description

We used the NSC (Koh et al., 2019) as our material to construct word frequency and contextual diversity norms for Singapore English. Initially, the NSC comprised three parts, each containing 1,000 h of English audio and transcripts from Singaporeans. In July 2021, it was updated with three additional parts, incorporating more varied speech scenarios.

The NSC currently consists of six parts: Part I contains phonetically balanced scripts; Part II includes randomly generated sentences; Part III comprises 1,000 h of transcripts from Singaporeans; Part IV features conversations where participants switch from English to their mother tongue; Part V contains content in which participants speak in four scenarios: debates, financial topics, positive emotions, and negative emotions; Part VI includes speech in three styles covering queries or conversations in daily scenarios (e.g., mobile plan renewal, at the restaurant, making inquiries with the Ministry of Education).

We selected Parts III, V, and VI for constructing our norms for the following reasons: (a) the content is primarily in English, and (b) these parts reflected naturalistic language production of Singaporean participants, reflecting authentic Singapore English usage patterns. To construct the word frequency and contextual diversity norms, we used all the available transcripts from three parts of the corpus: 911 from Part III (10,050,459 word tokens), 6,000 from Part V (10,343,605 word tokens), and 11,982 from Part VI (10,854,628 word tokens).

### Preprocessing

A Python script was used to clean the original TextGrid files. We extracted only the text property of each interval, as these represented the content spoken by participants in the conversations. After extraction, we removed punctuation, numbers, non-English characters, and text enclosed in angle brackets (< >), as these represented names and unintelligible words. All the words are converted to lowercase.

### Word frequency measures

We obtained a number of measures from the NSC—word frequency counts, Zipf values, and contextual diversity measures—closely following the methodology used in previous SUBTLEX databases (e.g., SUBTLEX-UK, van Heuven et al., 2014). This is an important point to mention because the consistent way in which these measures were computed can help researchers directly compare behavioral patterns and processing mechanisms between British/American English and Singapore English, minimizing the concern that observed differences are due to differences in how the frequency norms were derived.

#### Word frequency counts

For the word frequency counts, in line with the methodology used in SUBTLEX-UK (van Heuven et al., 2014), hyphenated words were separated into their individual components, with each component treated as a separate word type. For instance, the term “teh-o” was considered as two distinct word types: “teh” and “o.”

After cleaning the text, 56,679 word types with 31,248,692 word tokens remained, with each of the three parts containing over 10 million words. This represents a substantial corpus size for a less-studied English dialect, with the total word count approaching that of major frequency resources like SUBTLEX-US (~ 51 million words; Brysbaert et al., 2012). Most Singapore English dialect corpora contain fewer than 10 million tokens (Gonzales et al., 2023; Lin et al., 2023), making the NSC’s scale particularly valuable for robust frequency analysis of this variety. Word frequency counts reflect how often each word occurs in the corpus (see column “NSC_rawcount” in the CSV file provided on OSF).

#### Zipf values

In addition to the raw word count, we also provide a standardized measure of word frequency that allows for comparison across different frequency databases. The raw word count is heavily dependent on the corpus size, making a standardized value essential for cross-corpus comparisons. Compared to the more traditional frequency per million words (fpmw) measure, the Zipf scale is more suitable for corpora larger than one million words and is more sensitive for quantifying differences in raw word counts, better explaining word frequency effects (Brysbaert et al., 2018). Additionally, the Zipf scale allows for the inclusion of words with a frequency of zero (van Heuven et al., 2014). The Zipf value for the current corpus is calculated as follows (van Heuven et al., 2014):

$$Zipf=log_{10}\left(\frac{raw\;word\;frequency+1}{31.248692+0.05668}\right)+3$$

The denominator is the sum of the total word count and word types in millions. Consequently, words with a frequency of zero in the current SgE corpus have a Zipf value of 1.50. This value is higher than that in SUBTLEX-UK because the current corpus size is smaller than SUBTLEX-UK, resulting in less leverage from the corpus size for low-frequency words with zero occurrences. The Zipf measure can be found in column “NSC_Zipf.”

#### Contextual diversity

Contextual diversity measures the variety of contexts in which a word appears (Adelman et al., 2006). In the current study, we provide the raw count of documents in which the word occurs (“NSC_CDcount”) and the percentage of documents in the corpus in which the word is present (“NSC_CD”).

### Comparing the NSC with other English word frequency databases

**Fig. 1:**
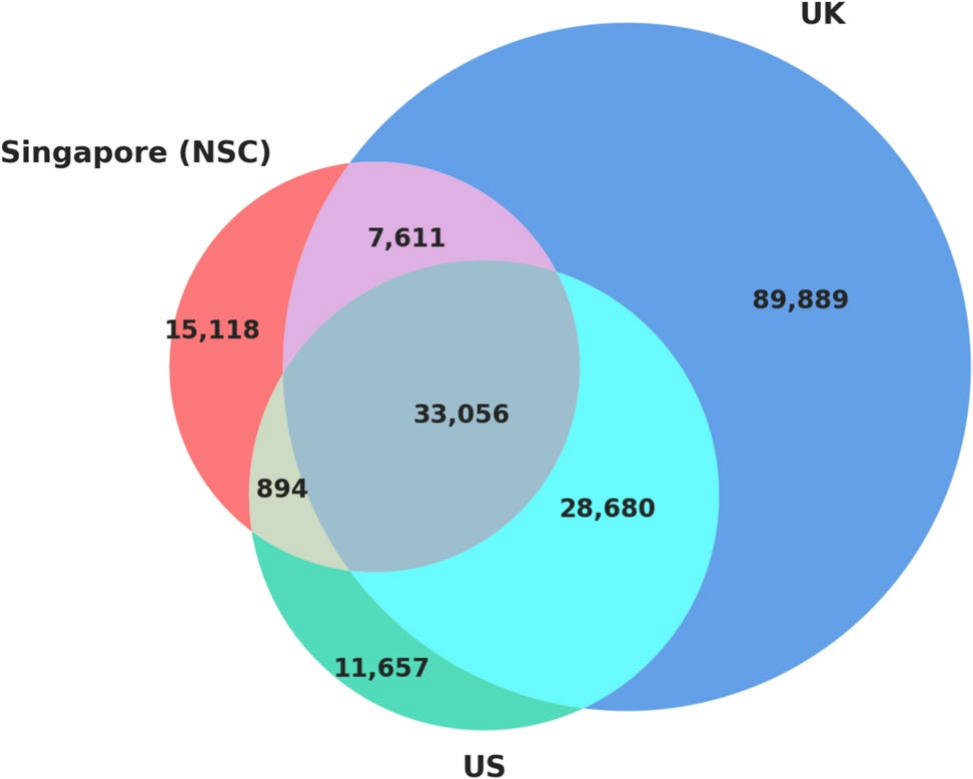
Venn diagram of the number of overlapping words between the databases

**Fig. 2:**
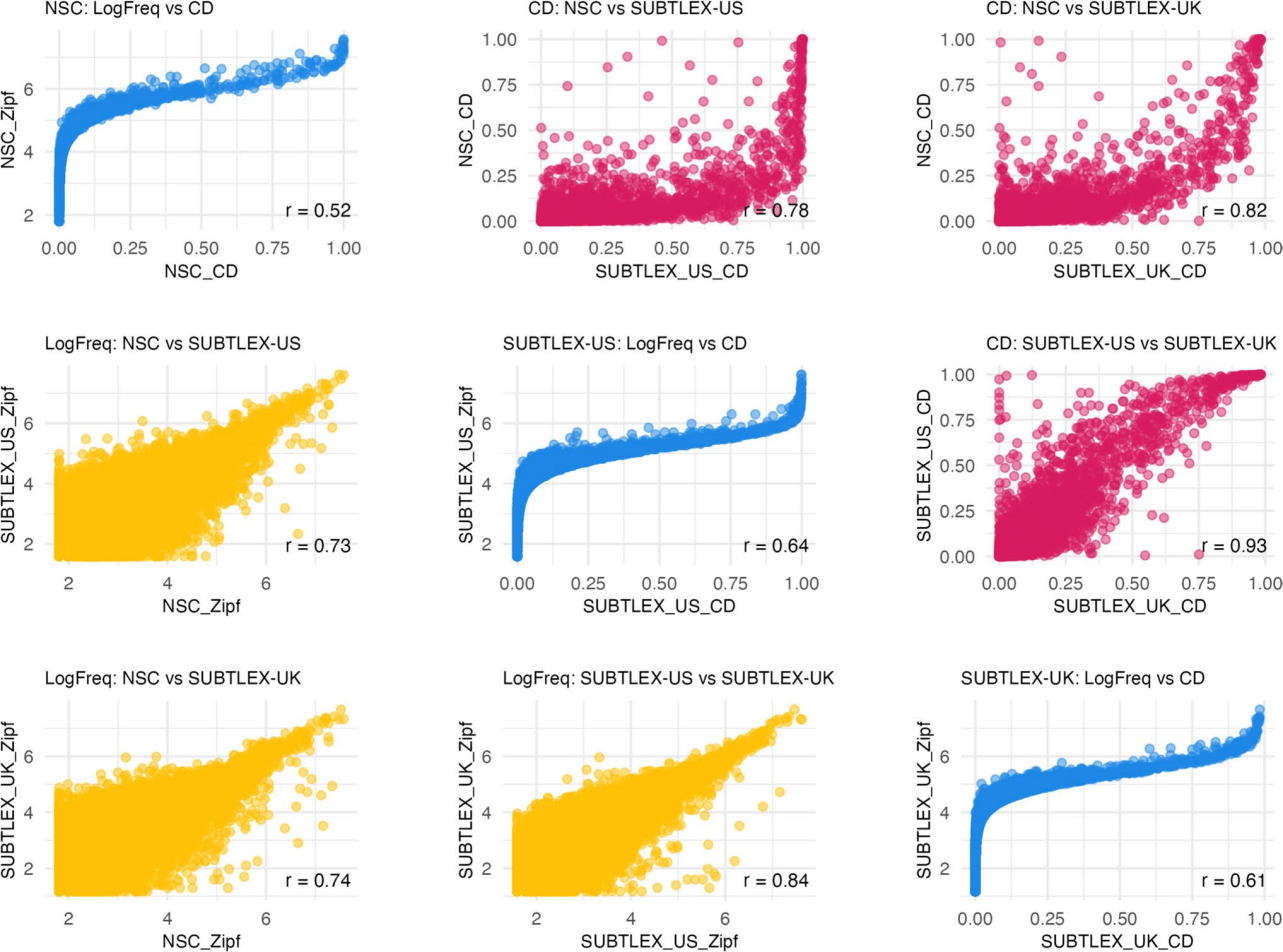
Scatterplots showing correlations of Zipf value and log_10_CD across databases

Comparing the NSC with the SUBTLEX-UK and SUBTLEX-US English frequency databases, we found 33,056 overlapping words across these three databases (see Fig. 1). In Fig. 2, we present scatterplots showing the relationships between Zipf values and log_10_(CD count) for each database pair. As evident from these plots, both NSC frequency and CD measures share significant large and positive correlations with the two SUBTLEX databases (all *p*s <.001).

As shown in Table 1, the NSC differs from both SUBTLEX-UK (van Heuven et al., 2014) and SUBTLEX-US (Brysbaert et al., 2012), though there is some overlap between them. The NSC, being a speech corpus, includes more frequent modal particles (e.g. um, eh, uh) compared to the other corpora. However, common patterns can be observed, especially for words with a Zipf value of 7 across all three corpora.

**Table 1:** Examples of words with similar and different Zipf values across NSC, SUBTLEX -UK (van Heuven et al., 2014), and SUBTLEX-US (Brysbaert et al., 2012)

	Same	Unique		
Zipf value (lower bound)		NSC	SUBTLEX-UK	SUBTLEX-US
1	sperms, ulla, elaborates, premade, cutted,…	floorboard, machiam, guetta, bazooka, grenades, …	doesn, singaporeans, yp, ite, leh, …	heartlands, tamil, neighboring, roti, natured,…
2	grasshopper, confiscate, menthol, phlegm, dalmatian, …	yps, yp, flashing, ew, laonua, …	craziest, drool, sia, legit, inconsiderate, …	acronym, maths, hostel, size, pioneering, …
3	folded, singer, jeans, stun, lavender,…	sharp, actor, ripped, rabak, unbelievable, …	okay, kay, twelve, ang, bro,…	nowadays, subjects, pets, vegetables, craziest, …
4	voice, jumping, corner, bunch, tree,…	orchard, aiyo, boy, piece, eyes,.	don, t, ya, yours, sign, …	alright, difference, near, eh, laundry …
5	hello, great, start, picture, called, …	alright, here, chicken, look, usually, …	low, mean, eh, famous, sure, …	ya, weird, which, ride, uh, …
6	my, why, think, let, with, …	t, um, lah, three, eh, …	than, ve, years, bit, into,…	okay, is, man, doing, sorry,…
7	I, that, s, it, of, you, a, the, and, to	okay, so, ya, like, for, uh	in	t

Table 2 shows the frequency of some common Singlish terms in the NSC. As a reminder, these are lexical items unique to Singapore English and do not exist in other dialects of English.

**Table 2:** Frequency and contextual diversity count of top 10 most frequent Singlish terms

Word	Meaning	Word count	Zipf value	CD
lah	a modal particle, usually used at the end of a sentence for a wide range of uses (e.g., reassurance, acceptance, speculation, softening)	139,858	6.65	9,678
orh	okay, to express acknowledgment or understanding	15,234	5.69	3,200
hdb	stands for “Housing and Development Board,” usually refers to public housing managed by the Housing and Development Board	11,852	5.59	2,003
leh	a modal particle, used at the end of a sentence to soften the tone	8,339	5.43	1,624
mah	a modal particle, used at the end of the sentence to express the self-evident	3,994	5.11	1,219
bto	stands for “Build-To-Order,” a scheme by HDB to build housing according to demand, where applicants apply for a new flat via a balloting system	3,507	5.05	870
sia	an exclamation that acts as an intensifier, adds coarseness	3,494	5.05	855
meh	a modal particle, used at the end of a negative question to ask for possibility	2,670	4.93	760
aiya	an exclamation of surprise or shock	2,488	4.90	1,098
loh	a modal particle that expresses resignation or reluctance	1,830	4.77	658

### Singapore English phonological neighborhood density (PND)

Singapore English has a distinct phonology compared to British and American English (Deterding, 2005; Goh et al., 2020; Low & Grabe, 1995); thus, PND based on British or American English might not be as applicable for Singapore English.

Our PND calculation employs the traditional definition: the number of words that differ from a target word by a single phoneme addition, deletion, or substitution (Luce & Pisoni, 1998). We construct a phonological network where words are connected if they are phonological neighbors, and PND is calculated as each word’s degree in this network (Vitevitch, 2008). This approach is methodologically identical to traditional PND calculations; the innovation lies in using Singapore English-specific phonological representations from the NSC’s IPA annotations rather than assuming British or American English phonologies.

Because some words in the NSC IPA annotation have multiple pronunciations, we constructed two types of networks:

1. Word-based network: In this network, nodes represent individual words, and edges connect words that share phonological similarities across their possible pronunciation variants. Words with multiple pronunciations have edges with other words based on all their pronunciations. For example, “act” (pronounced as “EH K” and “EH K T”) connects to both “a” (“EH” via deletion of “K” from “EH K”) and “actor” (“EH K T AA” via addition to “EH K T”).
2. Pronunciation-based network: In this network, nodes represent pronunciations, and edges connect nodes that are phonologically similar. Network measures for words with multiple pronunciations are averaged. For instance, the network measure for “act” is the average of the nodes “EH K” and “EH K T.”

From these two network models, we extracted neighborhood size (i.e., PND) for each word, which corresponded to the network measure known as *degree*. The correlation of PND between these two types of computation is 0.81 (*p* <.001). The word-based PND was used as a covariate in the main analysis reported in the next section. Word-based PND (“PND_word”) and pronunciation-based PND (“PND_pronoun”) are provided in the database.

For the number of phonemes, we computed the average across all pronunciations of a word. This value is included in the database as “number_of_phoneme.”

### Validation of the NSC norms: AELP analyses

To validate Singapore English frequency norms, we used data from the Auditory English Lexicon Project (AELP; Goh et al., 2020). This dataset is particularly suitable because it contains auditory lexical decision data from Singaporean participants. In contrast, several other available English megastudies (Balota et al., 2007; Keuleers et al., 2012; Tucker et al., 2019) contain behavioral data collected from predominantly North American or British participants. Additionally, the AELP presented auditory stimuli spoken by talkers with a Singaporean, American, or British English accent, enabling us to test the stability of the frequency effect across different accents.

## Method

### Data

We used auditory lexical decision data from the AELP (Goh et al., 2020), which collected trial-wise reaction time (RT) and accuracy (ACC) data from Singaporean participants who completed the auditory lexical decision task. The AELP consists of three English accents for the materials, American, British, and Singaporean, which were all recorded by two native speakers (one male and one female), respectively (i.e., a total of six different talkers). We analyzed the subset of data where trials were words which are mutually included in the NSC frequency, SUBTLEX-US (Brysbaert & New, 2009), and SUBTLEX-UK (van Heuven et al., 2014) databases.

Participants in the AELP (Goh et al., 2020) were recruited from the National University of Singapore community. All participants self-reported English as their first language, that they had no hearing or speech disorders, and that they had lived in Singapore for more than half of their lives.

### Covariates

For our analysis, we selected covariates based on Goh et al.’s (2020) original analysis of the AELP. The fixed-effect terms included token duration, NUS familiarity (Goh et al., 2020; the average subjective familiarity rating of words as reported by National University of Singapore students), prevalence (Brysbaert et al., 2019; an estimate of the percentage of the population familiar with a given word), number of phonemes (individual sound units that make up a word), and PND (i.e., phonological neighborhood size). For PND, because the same word in different accents can have different sets of phonological neighbors, for the data responding to the British and American accents, we used the PND values directly downloaded from the AELP (Goh et al., 2020), which calculated PND based on American and British phonological transcriptions, respectively. For the Singaporean accent, we calculated PND based on phonological transcriptions from the NSC (for details, please see previous section “Singapore English phonological neighborhood density”).

### Analytical strategy

We employed mixed-effects models to analyze the contribution of word frequency measures from each corpus in explaining participants’ data from the Auditory Lexical Decision Task (ALDT) of the AELP. For response time (RT) data, we used linear mixed-effects models using the lme4 package in R, while binomial mixed-effects models were applied to analyze accuracy data using the glmmTMB package (Brooks et al., 2017) in R.

Our models included fixed effects of token duration, number of phonemes, subjective familiarity, word prevalence, PND, and word frequency (Zipf-scaled). For the number of phonemes and PND, note that these measures were aligned to the corresponding accent (i.e., the Singapore English PND measure was used as a covariate when analyzing the Singaporean accent data, whereas the American English PND measure was used as a covariate when analyzing the American accent data).

The random effect’s structure comprised random intercepts for participants and items. The baseline model included all control variables, while each corpus-specific model (NSC, SUBTLEX-US, and SUBTLEX-UK) replaced the baseline frequency measure with its respective corpus frequency or contextual diversity measure.

To compare the fit of the frequency and CD norms, we used the Akaike information criterion (AIC, Akaike, 1974) to assess model fit, and employed Akaike weights (Wagenmakers & Farrell, 2004) to quantify the relative support for the best-fitting corpus compared to alternatives. We used the “qpcR” package in R to calculate the Akaike weights (Spiess, 2018). For each accent group, we summed the Akaike weights of both the word frequency and contextual diversity models for each corpus separately, analyzing RT and accuracy data independently. For example, to compare the relative support for NSC versus SUBTLEX-US in explaining RT data within a specific accent group of models, we calculated

$$\frac{{w}_{{RT}_{{NSC}_{wfreq}}}\left(AIC\right)+{w}_{{RT}_{{NSC}_{CD}}}\left(AIC\right)}{{w}_{{RT}_{{SUBTLEX-US}_{wfreq}}}\left(AIC\right)+{w}_{{RT}_{{SUBTLEX-US}_{CD}}}\left(AIC\right)}$$

In addition to AIC, we reported the marginal *R*^2^ of each model, which estimates the variance explained by the fixed effects. It should be noted that marginal *R*^2^ is not directly comparable to the *R*^2^ obtained from standard linear regression models (Roback & Legler, 2021). We include these values to provide readers with a fuller picture of model performance by indicating the proportion of variance explained by the fixed effects in our mixed-effects models.

Given our focus on cross-accent comparisons, we only examined words with consistent spellings across American and British English. Words were excluded based on two criteria: (a) words marked as having variant spellings in the AELP, and (b) words with potential spelling variations not explicitly marked in the AELP based on the *breame* package (e.g., “airplane”/“aeroplane,” “cancellation”/“cancelation” Charles, 2025). This screening process resulted in the exclusion of 253 words.

We also excluded words which did not have a familiarity rating and prevalence rating, two of the covariates in the models. This led to the exclusion of 979 words. Finally, we only analyzed trials with words that mutually existed in all three databases, resulting in the exclusion of 634 words.

For the Singaporean accent analysis, 287 words were excluded due to missing IPA transcriptions in the NSC database, which prevented us from computing their PND values. These words were retained in the British and American accent analyses. As a result, the British and American accents each included 8,305 words, whereas the Singaporean accent analysis included 8,018 words.

Reaction time (RT) analysis only included the trials that were analyzed in the original AELP study (Goh et al., 2020), which excluded RT trials if the response was incorrect, if the RT was outside the range of 200 ms to 3,000 ms, or if the RT fell beyond 2.5 *SD*s from the participant’s mean RT. No additional filtering criteria were applied for accuracy analysis.

## Results

### Singaporean accent

Tables 3 and 4 demonstrate that both word frequency (Zipf value) and contextual diversity of NSC were significantly associated with faster response times and higher accuracy when participants responded to Singaporean-accented words.

**Table 3:** Linear mixed-effects model results for the effects of Zipf value and contextual diversity from the National Speech Corpus on response time in the Singaporean Accent Auditory Lexical Decision Task (Goh et al., 2020). CD = contextual diversity; PND = phonological neighborhood density; *SE* = standard error. Both PND and number of phonemes are based on Singaporean accent

	Response time (linear mixed-effects model)			
	Word frequency		Contextual diversity	
Predictors	Estimates	SE	Estimates	SE
(Intercept)	1,103.36^***^	17.25	1,006.59^***^	19.76
Duration	0.55^***^	0.00	0.55^***^	0.00
Number of phonemes	− 7.84^***^	0.50	− 7.86^***^	0.50
Familiarity	− 56.42^***^	2.65	− 54.88^***^	2.67
Prevalence	− 44.00	3.93	− 43.67^***^	3.93
PND	0.82^***^	0.04	0.82^***^	0.04
**NSC Zipf value**	− 13.86^***^	**0.99**		
**Log**_**10**_**(CD_NSC)**			− 14.88^***^	**1.04**
**Random effects**				
σ^2^	47,152.88		47,152.91	
τ_00_	4,133.89 _word_		4,127.47 _word_	
	7,044.73 _subject_		7,044.05 _subject_	
Marginal *R*^2^	0.162		0.162	

*Note:* *** <.001; ** <.01; * <.05

**Table 4:** Generalized linear mixed-effects model results for the effects of Zipf value and contextual diversity from the National Speech Corpus on accuracy in the Singaporean Accent Auditory Lexical Decision Task (Goh et al., 2020). CD = contextual diversity; PND = phonological neighborhood density. Both PND and number of phonemes are based on Singaporean accent

	Accuracy (generalized linear mixed-effects model)			
	Word frequency		Contextual diversity	
Predictors	Odds ratios	SE	Odds ratios	SE
(Intercept)	0.00^***^	0.00	0.01^***^	0.00
Duration	1.00^***^	0.00	1.00^***^	0.00
Number of phonemes	1.24^***^	0.01	1.24^***^	0.01
Familiarity	2.82^***^	0.11	2.74^***^	0.10
Prevalence	2.14^***^	0.13	2.12^***^	0.13
PND	0.99^***^	0.00	0.99^***^	0.00
**NSC Zipf Value**	1.24^***^	**0.02**		
**Log**_**10**_**(CD_NSC)**			1.27^***^	0.02
**Random effects**				
σ^2^	3.29		3.29	
τ_00_	0.39 _subject_		0.39 _subject_	
	0.90 _word_		0.90 _word_	
Marginal *R*^2^	0.121		0.121	

*Note:* *** <.001; ** <.01; * <.05

**Fig. 3:**
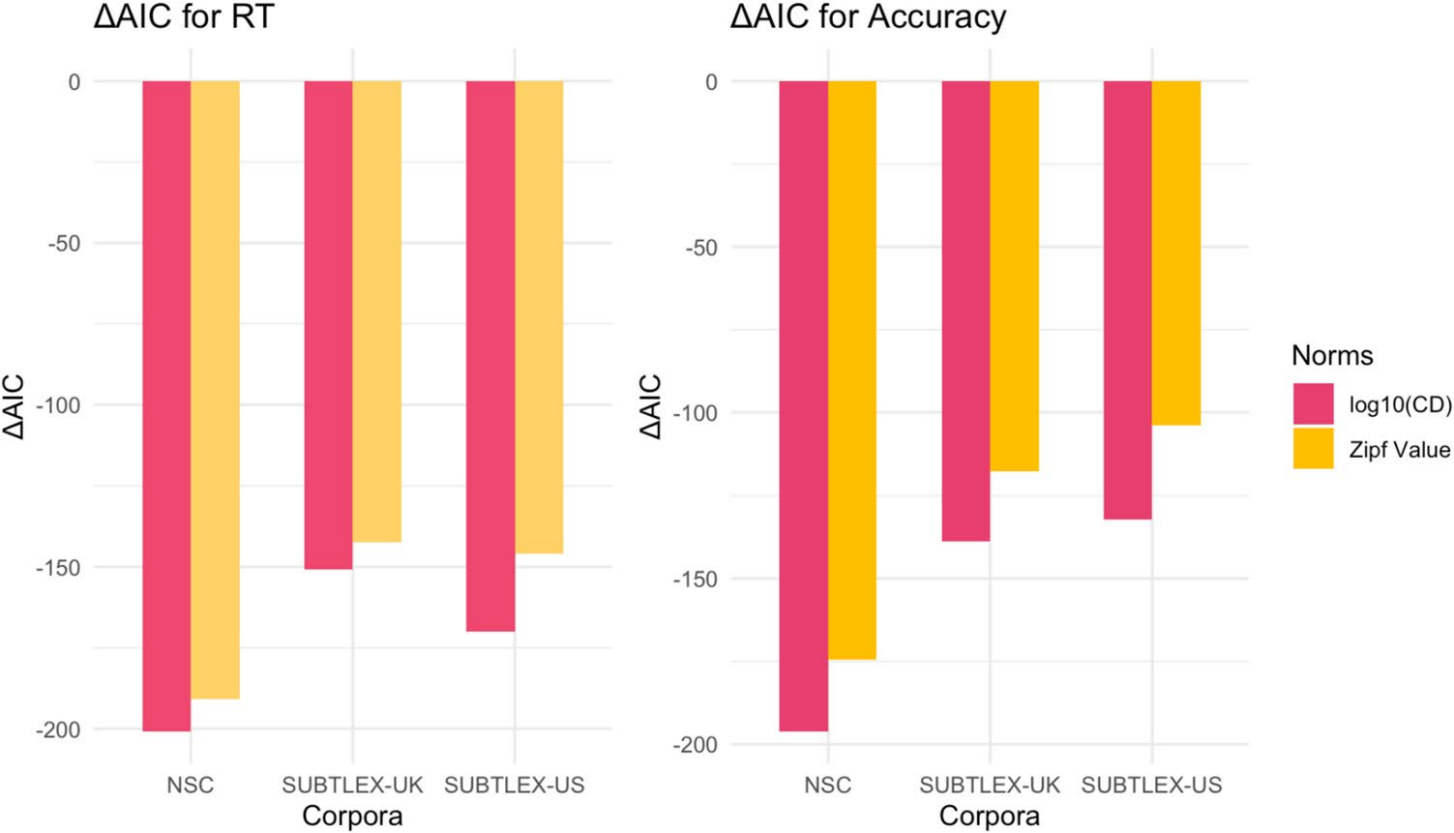
Comparison of model fit using different frequency and contextual diversity measures on response time (RT) and accuracy (ACC) for Singaporean Accent in the Auditory English Lexicon Project (AELP; Goh et al., 2020). AIC values for mixed-effects models incorporating frequency or contextual diversity measures from various corpora. All models included fixed effects for duration, number of phonemes, familiarity, and prevalence, phonological neighborhood density, with participant and word as random effects (*df*_baseline_ = 9). CD = contextual diversity

Figure 3 shows that frequency and contextual diversity measures from all three corpora reduced the AIC compared to the baseline model in reaction time analysis. Contextual diversity consistently produced greater AIC reductions than Zipf value across all corpora. The NSC showed the largest AIC reductions for both contextual diversity and frequency measures, relative to the same measures from SUBTLEX-UK and SUBTLEX-US. When comparing the models using Akaike weights (combining both contextual diversity and word frequency for each corpus), the NSC model was 4.61 × 10⁶ times more likely to be the best model for reaction time than the second-best model (SUBTLEX-US). For accuracy data, the NSC model was 2.58 × 10^12^ times more likely to be the best model than the second-best model (SUBTLEX-UK). These results strongly suggest that the NSC provides a superior fit for modeling both reaction time and accuracy data in Singaporean-accented auditory lexical decision task.

### British accent

Tables 5 and 6 show that for British-accented words, higher NSC word frequency and contextual diversity values were significantly associated with faster response times and higher accuracy.

**Table 5:** Linear mixed-effects model results for the effects of Zipf value and contextual diversity from the National Speech Corpus on response time in the British Accent Auditory Lexical Decision Task (Goh et al., 2020). CD = contextual diversity; PND = phonological neighborhood density. Both PND and number of phonemes are based on British accent (Goh et al., 2020)

	Response time (linear mixed-effects model)			
	Word frequency		Contextual diversity	
Predictors	Estimates	SE	Estimates	SE
(Intercept)	1,032.39^***^	16.55	922.74^***^	19.18
Duration	0.52^***^	0.00	0.52^***^	0.00
Number of phonemes	− 3.10^***^	0.53	− 3.11^***^	0.53
Familiarity	− 45.44^***^	2.58	− 43.78^***^	2.61
Prevalence	− 53.50^***^	4.06	− 53.12^***^	4.06
PND	3.60^***^	0.14	3.59^***^	0.14
**NSC Zipf value**	− 15.83^***^	**1.03**		
**Log**_**10**_**(CD_NSC)**			− 16.89^***^	**1.08**
**Random effects**				
σ^2^	47,662.75		47,662.76	
τ_00_	4,829.39 _word_		4,821.65 _word_	
	6,937.02 _subject_		6,937.55 _subject_	
Marginal *R*^2^	0.124		0.124	

*Note:* *** <.001; ** <.01; * <.05

**Table 6:** Generalized linear mixed-effects model results for the effects of Zipf value and contextual diversity from the National Speech Corpus on accuracy in the British Accent Auditory Lexical Decision Task (Goh et al., 2020). CD = contextual diversity; PND = phonological neighborhood density. Both PND and number of phonemes are based on British accent (Goh et al., 2020)

	Accuracy (generalized linear mixed-effects model)			
	Word frequency		Contextual diversity	
Predictors	Odds ratios	SE	Odds ratios	SE
(Intercept)	0.00^***^	0.00	0.01^***^	0.00
Duration	1.00^***^	0.00	1.00^***^	0.00
Number of phonemes	1.26^***^	0.01	1.26^***^	0.01
Familiarity	2.50^***^	0.09	2.42^***^	0.09
Prevalence	2.54^***^	0.16	2.52^***^	0.16
PND	0.98^***^	0.00	0.98^***^	0.00
**NSC Zipf value**	1.34^***^	**0.02**		
**Log**_**10**_**(CD_NSC)**			1.37^***^	**0.02**
**Random effects**				
σ^2^	3.29		3.29	
τ_00_	0.47 _subject_		0.47 _subject_	
	1.00 _word_		1.00 _word_	
Marginal *R*^2^	0.135		0.135	

*Note:* *** <.001; ** <.01; * <.05

**Fig. 4:**
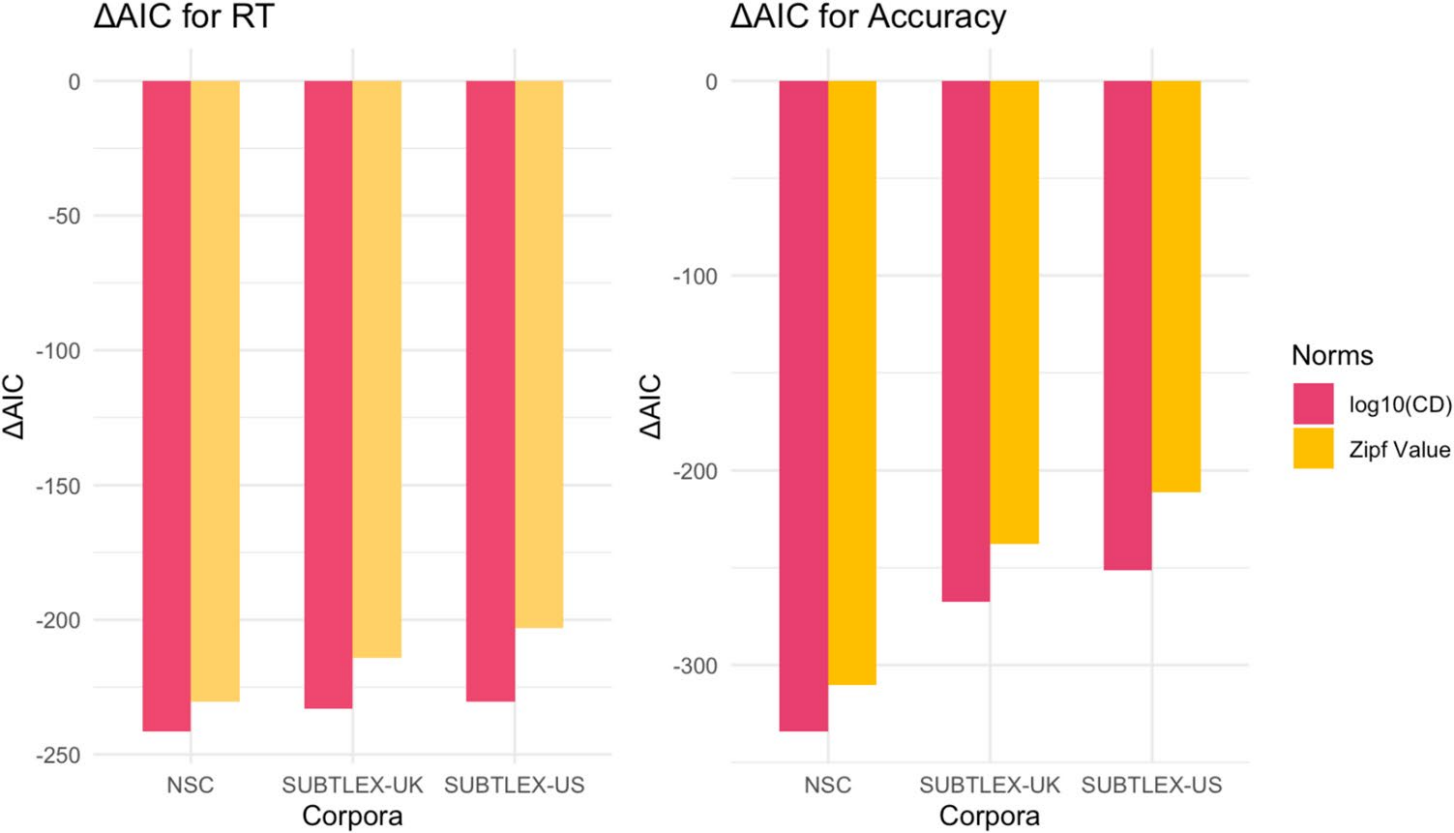
Comparison of model fit using different frequency and contextual diversity measures on response time (RT) and accuracy (ACC) for British Accent in the Auditory English Lexicon Project (AELP; Goh et al., 2020). AIC values for mixed-effects models incorporating frequency or contextual diversity measures from various corpora. All models included fixed effects for duration, number of phonemes, familiarity, and prevalence, phonological neighborhood density, with participant and word as random effects (*df*_baseline_ = 9). CD = contextual diversity

Aligning with our findings for Singaporean-accented speech, Fig. 4 reveals that measures from all three corpora reduced the AIC compared to the baseline model, with contextual diversity producing larger reductions than Zipf value. The NSC measures again showed the greatest reduction among all corpora. Analysis of Akaike weights (combining contextual diversity and word frequency) showed that for reaction time, the NSC model was 68.29 times more likely to be the better model than SUBTLEX-UK. For accuracy, the NSC model was 2.50 × 10^14^ times more likely to be optimal than SUBTLEX-UK. These findings further support NSC’s superior fit for modeling Singaporeans’ language experience, extending from Singaporean- to British-accented speech.

### American accent

Tables 7 and 8 show that higher NSC word frequency and contextual diversity values were significantly correlated with faster response times and higher accuracy for Singaporean participants responding to American-accented words.

**Table 7:** Linear mixed-effects model results for the effects of Zipf value and contextual diversity from the National Speech Corpus on response time in the American Accent Auditory Lexical Decision Task (Goh et al., 2020). CD = contextual diversity; PND = phonological neighborhood density. Both PND and number of phonemes are based on American accent (Goh et al., 2020)

	Response time (linear mixed-effects model)			
	Word frequency		Contextual diversity	
Predictors	Estimates	SE	Estimates	SE
(Intercept)	1,141.84^***^	16.48	1,037.17^***^	18.96
Duration	0.41^***^	0.00	0.41^***^	0.00
Number of phonemes	2.22^***^	0.51	2.20^***^	0.51
Familiarity	− 47.01^***^	2.49	− 45.36^***^	2.52
Prevalence	− 59.78^***^	3.94	− 59.33^***^	3.93
PND	3.39^***^	0.15	3.38^***^	0.15
**NSC Zipf value**	− 14.95^***^	1.00		
**Log**_**10**_**(CD_NSC)**			− 16.08^***^	1.04
**Random effects**				
σ^2^	49,358.49		49,358.53	
τ_00_	4,642.23 _word_		4,632.87 _word_	
	9,638.15 _subject_		9,639.14 _subject_	
Marginal *R*^2^	0.074		0.074	

*Note:* *** <.001; ** <.01; * <.05

**Table 8:** Generalized linear mixed-effects model results for the effects of Zipf value and contextual diversity from the National Speech Corpus on accuracy in the American Accent Auditory Lexical Decision Task (Goh et al., 2020). CD = contextual diversity; PND = phonological neighborhood density. Both PND and number of phonemes are based on American accent (Goh et al., 2020)

	Accuracy (generalized linear mixed-effects model)			
	Word frequency		Contextual diversity	
Predictors	Odds ratios	SE	Odds ratios	SE
(Intercept)	0.00^***^	0.00	0.00^***^	0.00
Duration	1.00^*^	0.00	1.00^*^	0.00
Number of phonemes	1.22^***^	0.01	1.22^***^	0.01
Familiarity	2.58^***^	0.10	2.50^***^	0.10
Prevalence	2.90^***^	0.19	2.86^***^	0.18
PND	0.98^***^	0.00	0.98^***^	0.00
**NSC Zipf value**	1.29^***^	**0.02**		
**Log**_**10**_**(CD_NSC)**			1.32^***^	**0.02**
**Random effects**				
σ^2^	3.29		3.29	
τ_00_	0.48 _subject_		0.48 _subject_	
	1.13 _word_		1.12 _word_	
Marginal *R*^2^	0.127		0.128	

*Note:* *** <.001; ** <.01; * <.05

**Fig. 5:**
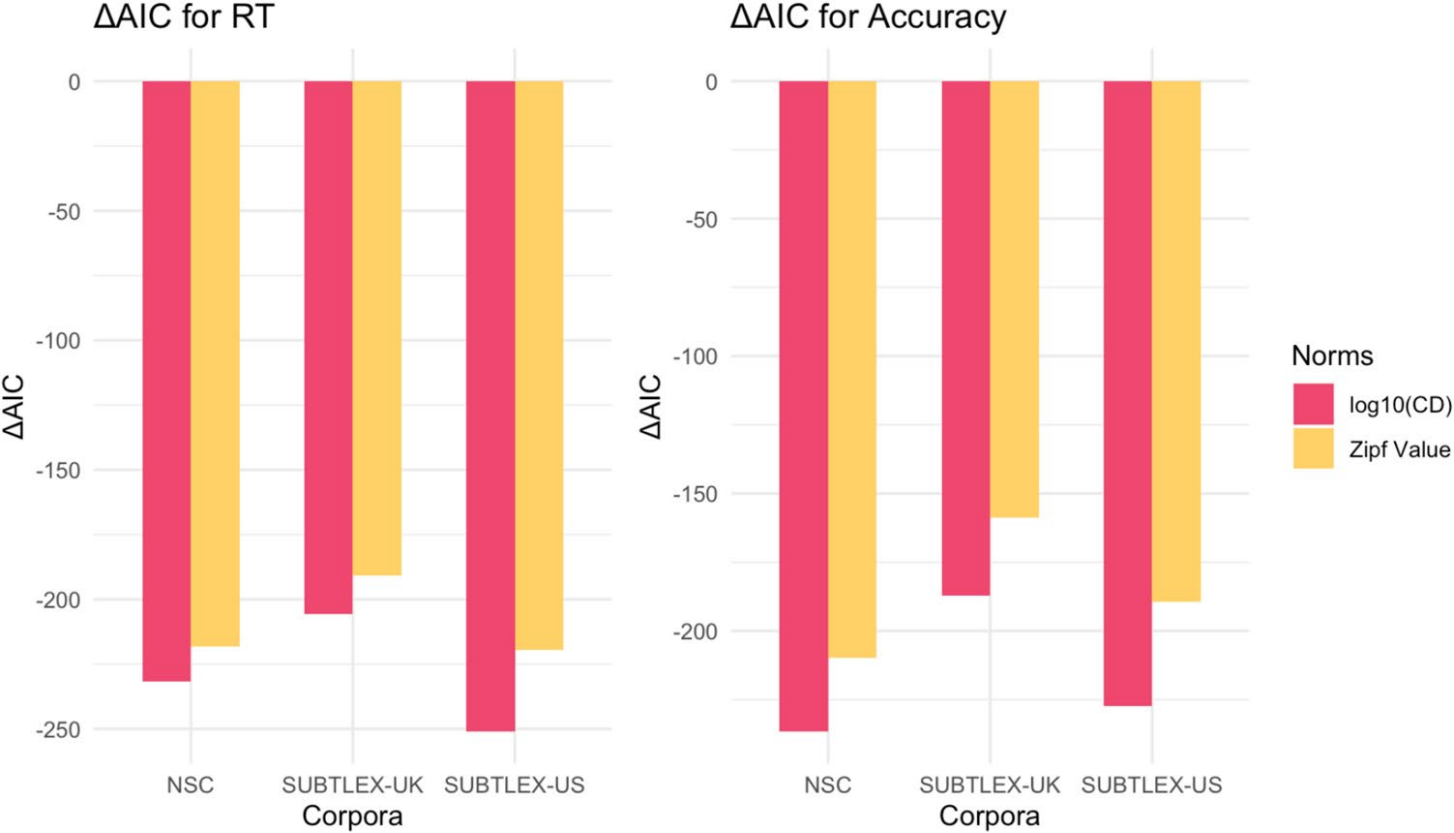
Comparison of model fit using different frequency and contextual diversity measures on response time (RT) and accuracy (ACC) for American Accent in Auditory English Lexicon Project (AELP; Goh et al., 2020). AIC values for mixed-effects models incorporating frequency or contextual diversity measures from various corpora. All models included fixed effects for duration, number of phonemes, familiarity, and prevalence, phonological neighborhood density, with participant and word as random effects (*df*_baseline_ = 9)

Unlike our findings for Singaporean and British accents, Fig. 5 reveals that SUBTLEX-US measures showed the largest AIC reduction for reaction time, although NSC remained the best model for accuracy. Analysis of Akaike weights (combining contextual diversity and word frequency) showed that for reaction time, the SUBTLEX-US model was 1.33 × 10^4^ times more likely to be optimal than the second-best model (NSC). For accuracy, however, the NSC model was 88.61 times more likely to be the better model than SUBTLEX-US. These findings suggest a distinctive pattern for American-accented speech, where SUBTLEX-US performed better in predicting reaction time while NSC performed better in predicting accuracy.

## General discussion

Word frequency norms are essential tools in psycholinguistic research, providing quantitative measures of how often words occur in a language. However, previous popular word norms have predominantly focused on mainstream English varieties (Brysbaert et al., 2012; van Heuven et al., 2014), overlooking dialectal variations that can lead to potential misinterpretations and inaccurate cognitive predictions when applied to specific English dialects. The NSC word frequency and contextual diversity norms represent a significant contribution as the largest database derived from Singapore English speech to date. Compared to previous Singapore English frequency databases (Greenbaum, 1991; Lin et al., 2023; Siew, 2024), the current database offers unprecedented scale and is constructed directly from authentic speech samples, providing a more accurate reflection of Singaporeans’ linguistic environment. Through a comparative analysis of auditory lexical decision data across different English accents, we demonstrated that the NSC measures outperform both American and British English measures when processing words spoken in Singaporean and British accents, validating its effectiveness as a representative word frequency database for psycholinguistic research conducted on Singapore English. The NSC offers unique advantages by being derived from natural speech, which can effectively capture the distinctiveness of Singapore English, particularly its colloquial elements. Additionally, it provides frequency and contextual diversity estimates for Singapore-specific terms such as “hdb” (Housing and Development Board) and “bto” (Build-To-Order), which are concepts commonly used in Singaporean daily life but absent from other English variants. This work not only establishes NSC as a valid representation of language usage patterns for Singapore English but also demonstrates the feasibility of using speech corpora to estimate word frequency norms for various English dialects. This highlights a broader issue in psycholinguistics—word frequency norms must be tailored to specific linguistic communities to accurately capture their language processing patterns.

Building on this idea, the study of diverse English dialects is crucial for advancing our understanding of language processing. Word frequency effects reflect individuals’ language exposure (Brysbaert et al., 2018), and numerous studies have demonstrated that these effects are better explained by the specific language variant that speakers regularly encounter (Siew, 2024; van Heuven et al., 2014). Our research provides compelling evidence for the necessity of constructing word frequency databases based on specific dialects rather than relying solely on dominant variants of English. While different English variants share many similarities, they can vary substantially in grammatical structures and semantics (Bao et al., 1998; Wong, 2004). Singapore-specific terms such as “hdb” and “bto” do not exist in American or British English, despite having concrete meanings within Singaporean culture. Since word knowledge varies from individual to individual (Johns, 2023), research analyzing specific populations must incorporate language statistics that accurately reflect those populations’ linguistic experiences. Furthermore, the predominance of “standard” English in cognitive science has raised questions about the generalizability of results across diverse linguistic communities (Blasi et al., 2022; Henrich et al., 2010; Kirk, 2023; Kutlu & Hayes-Harb, 2023; Levisen, 2019). By studying dialect variations systematically, we can address these limitations, enhance the cross-cultural validity of psycholinguistic models, and develop more inclusive approaches to language research that acknowledge the rich diversity of English worldwide.

Interestingly, in the current study, we found that the NSC measures did not outperform SUBTLEX-US when participants processed words spoken in the American accent. This result contrasts with their performance in the other two accents, and we speculate that this may be attributed to Singaporeans’ mixed linguistic exposure, particularly given the pervasive global influence of American English. Globalization has reinforced American influence on Singaporean English through pop culture and institutional preferences (Canan Hänsel & Deuber, 2013; Lee, 2001; Pew Research Center, 2012), which likely contribute to heightened familiarity with American English among Singaporeans.

As a result, exposure to American English may be more deeply embedded in Singaporeans’ linguistic experience, potentially making the American-accented stimuli function as a meta-linguistic prime in our study. Prior research on interpersonal communication has suggested that accents help listeners construct speaker profiles, which in turn facilitates lexical prediction (Cai et al., 2017). The American accent may have activated participants’ language experience associated with American English, making the corresponding word frequencies more relevant during lexical processing. In contrast, the British accent did not serve as an effective prime, possibly because British cultural influence in Singapore is weaker than that of the United States. Additionally, British pronunciation shares greater phonetic similarity with Singapore English (Goh et al., 2020), which may have made it less distinctive as a meta-linguistic prime. These results suggest flexibility in language processing among speakers who have varied exposure to different dialects of the same language, who can switch language representations in response to phonological cues. This indicates the possible existence of multiple language systems in the mind that dynamically activate during daily usage to better process linguistic stimuli in the environment, even when these systems belong to the same language category (English in this case). While this pattern resembles what has been observed among Scots speakers (Kirk, 2023), it is important to note that Singaporeans are predominantly multilingual, with over 70% of the population speaking multiple languages (Ong, 2021). This multilingualism likely introduces additional complexity to the language processing mechanisms of Singapore English speakers. Though beyond the scope of the current study, this phenomenon merits further investigation in future research. These findings underscore the critical importance of developing dialect-specific tools such as our NSC frequency database, as such resources enable researchers to accurately capture the unique linguistic processing patterns of speakers who speak nondominant varieties or dialects that would otherwise be obscured when using mainstream English measures.

Another significant finding in our study is the consistent superiority of contextual diversity measures over word frequency measures across all three accents and databases, which aligns with previous research (Adelman & Brown, 2008; Adelman et al., 2006; Boada et al., 2020; Brysbaert & New, 2009; van Heuven et al., 2014).While word frequency primarily reflects raw counts of occurrence, which may not be directly related to lexical representation, contextual diversity captures the distinctiveness of word usage across varied linguistic environments. Johns (2021) suggested that contextual diversity corresponds to the *principle of likely need* in memory models—as words appear in more diverse contexts, they become more likely to be relevant in any new context, thus increasing their cognitive availability. This finding reinforces the importance of incorporating contextual diversity measures in psycholinguistic research to more accurately predict language processing patterns.

Our study found lower variance explained by word frequency than previous studies reporting 30.7–42.7% (Balota et al., 2004; Keuleers et al., 2010b, 2012; Spieler & Balota, 1997) as well as the original AELP analysis (Goh et al., 2020). This discrepancy reflects methodological differences: previous studies used item-level linear regression, while we employed trial-level mixed-effects models. The marginal *R*^2^ from mixed-effects models represents variance explained by fixed effects alone and is not directly comparable to standard linear regression *R*^2^ values (Roback & Legler, 2021).

It should also be noted that the current database does not include part-of-speech (PoS) information, which represents a limitation of this resource. Singapore English contains numerous informal and nonstandard linguistic features that present challenges for automated PoS tagging (Chan & Ng, 2024; Huang et al., 2025; Lin et al., 2023). While several studies have attempted to apply the standard PoS taggers for Singapore English, achieving varying degrees of success, the accuracy remains lower than that of PoS tagging tools designed for standard varieties of English, particularly when processing informal usage patterns (Chan & Ng, 2024; Lin et al., 2023). Given that this database is intended for generalized psycholinguistic tasks, we prioritized maintaining accuracy levels comparable to other established databases rather than including potentially unreliable PoS annotations. We encourage future research to develop more robust off-the-shelf PoS tagging tools specifically designed for Singapore English and other understudied English varieties, which would enable the inclusion of accurate PoS information in subsequent versions of this database.

Overall, this study addresses a critical gap in psycholinguistic research by developing the first comprehensive word frequency database specifically tailored to Singapore English. Our findings demonstrate that linguistic processing is fundamentally shaped by dialectal variation, challenging the assumption that mainstream English norms can adequately represent all English speakers’ experiences. The NSC database not only provides researchers with a powerful new tool for investigating Singapore English but also establishes the importance for developing similar resources for other English varieties worldwide. By capturing the unique linguistic patterns of Singapore English—including its distinctive vocabulary, contextual usage, and cultural references—this database enables more accurate modeling of language processing in this significant English-speaking community. Beyond its immediate applications in psycholinguistic research, our work underscores the broader importance of linguistic diversity in cognitive science and the need to recognize dialectal variation as central rather than peripheral to our understanding of language processing. As global communication continues to evolve, such dialect-specific resources will become increasingly essential for developing inclusive language technologies, educational practices, and theoretical models that truly reflect the rich tapestry of English as it is actually used across the world.

## Supplementary Information

Below is the link to the electronic supplementary material.

Supplementary file1 (DOCX 151 KB)

## Data Availability

The data and materials reported in this paper can be found at: https://osf.io/ku4p9

## Code availability

The analysis code used in this paper can be found at https://osf.io/ku4p9

## Declarations

### Open practices statement

None of the analyses were preregistered. Word frequency and contextual diversity measures and analysis scripts for the results reported in this paper can be found at https://osf.io/ku4p9.
