# Supplementary material for: Word frequency and contextual diversity measures for Singapore English
Source: Behav Res Methods. 2026 Apr 29;58(6):149. doi: 10.3758/s13428-026-03012-1 (PMC13128781; doi:10.3758/s13428-026-03012-1)
Supplement: Supplementary file 1 — Supplementary file1 (DOCX 151 KB) [file 13428_2026_3012_MOESM1_ESM.docx]

# Supplementary Materials

Word frequency and contextual diversity measures for Singapore English

[Analysis with the Singapore English (SgE) phonological neighborhood density (PND) for both American and British accented auditory lexical decision task 2](#_Toc256342452)

[Model summaries with the SUBTLEX-US and SUBTLEX-UK for Singaporean, British and American accent in the manuscript. 13](#_Toc495394882)

## Analysis with the Singapore English (SgE) phonological neighborhood density (PND) for both American and British accented auditory lexical decision task

Analyses using Singaporean English (SgE) PND and number of phonemes as covariates in the British- and American-accented auditory lexical decision tasks yielded results consistent with those reported in the main manuscript. In the British-accented task, the National Speech Corpus (NSC) measures outperformed the alternatives, while in the American-accented task, NSC also generally showed better performance. The only deviation concerned accuracy in the American-accented task: when using American PND, SUBTLEX-US performed worse than NSC, whereas with Singaporean PND, SUBTLEX-US performed better. The Akaike weights further indicated that the model incorporating SUBTLEX-US contextual diversity was 1.33 times more likely to be the best-fitting model compared with the one using NSC. Tables S1–S12 provide detailed model summaries.


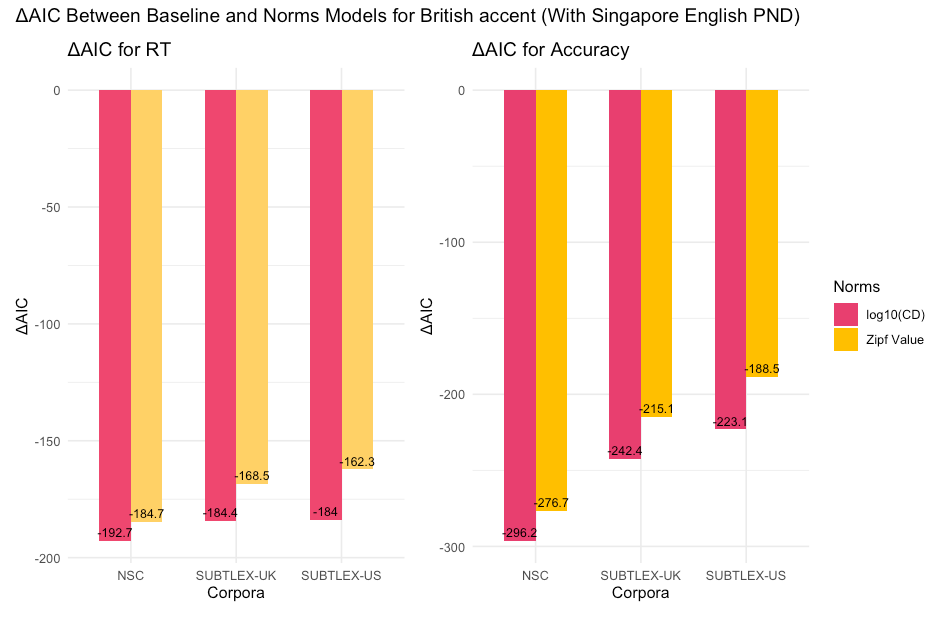


**Figure S 1** Comparison of Model Fit Using Different Frequency and Contextual Diversity Measures on Response time (RT) and Accuracy (ACC) for British Accent in Auditory English Lexicon Project (AELP, Goh et al., 2020) with Singapore English phonological neighborhood density and number of phonemes. AIC values for mixed-effects models incorporating frequency or contextual diversity measures from various corpora. All models included fixed effects for duration, number of phonemes, familiarity, and prevalence, phonological neighborhood density, with participant and word as random effects.(df_baseline_ = 9). CD = contextual diversity


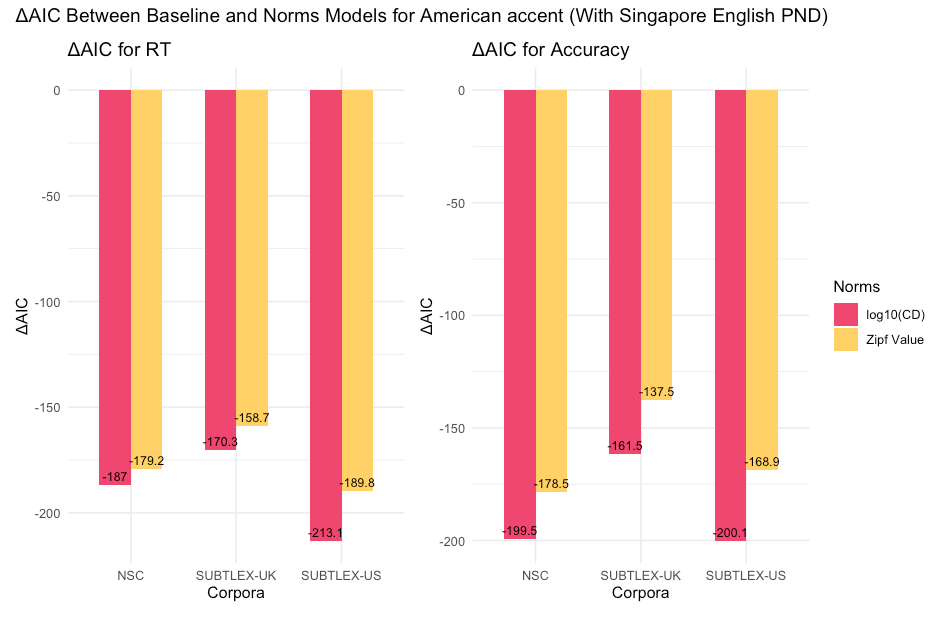


**Figure S 2** Comparison of Model Fit Using Different Frequency and Contextual Diversity Measures on Response time (RT) and Accuracy (ACC) for American Accent in Auditory English Lexicon Project (AELP, Goh et al., 2020) with Singapore English phonological neighborhood density and number of phoneme. AIC values for mixed-effects models incorporating frequency or contextual diversity measures from various corpora. All models included fixed effects for duration, number of phonemes, familiarity, and prevalence, phonological neighborhood density, with participant and word as random effects.(df_baseline_ = 9). CD = contextual diversity

**Tabe S 1** Linear mixed-effects model results for the effects of Zipf Value and Contextual Diversity from NSC on Response Time in the British Accent Auditory Lexical Decision Task (Goh et al., 2020). CD = Contextual Diversity; PND = Phonological Neighborhood Density; SE = Standard Error. Both PND and number of phonemes are based on Singaporean accent.

|  | *Response Time(linear mixed effect model)* | | | |
| --- | --- | --- | --- | --- |
|  | *Word frequency* | | *Contextual Diversity* | |
| *Predictors* | *Estimates* | *SE* | *Estimates* | *SE* |
| (Intercept) | 1072.84 | 18.02 | 971.38 | 20.75 |
| Duration | 0.52^***^ | 0.00 | 0.52^***^ | 0.00 |
| Number of phonemes | -5.28^***^ | 0.53 | -5.29^***^ | 0.53 |
| Familiarity | -49.14^***^ | 2.82 | -47.57^***^ | 2.85 |
| Prevalence | -53.91^***^ | 4.22 | -53.62^***^ | 4.22 |
| PND | 0.87^***^ | 0.04 | 0.86^***^ | 0.04 |
| **NSC Zipf Value** | **-14.62^***^** | **1.06** |  |  |
| **Log_10_(CD_NSC)** |  |  | **-15.64**^***^ | **1.11** |
| **Random Effects** | |  |  |  |
| σ^2^ | 47419.42 |  | 47419.43 |  |
| τ_00_ | 4923.39 _word_ |  | 4917.51 _word_ |  |
|  | 6876.89 _subject_ |  | 6877.15 _subject_ |  |
| Marginal R^2^ | 0.120 |  | 0.120 |  |

*Note: ***, <.001; **,<.01; *, <.05*

**Tabe S 2** Linear mixed-effects model results for the effects of Zipf Value and Contextual Diversity from SUBTLEX-UK on Response Time in the British Accent Auditory Lexical Decision Task (Goh et al., 2020). CD = Contextual Diversity; PND = Phonological Neighborhood Density; SE = Standard Error. Both PND and number of phonemes are based on Singaporean accent.

|  | *Response Time(linear mixed effect model)* | | | |
| --- | --- | --- | --- | --- |
|  | *Word frequency* | | *Contextual Diversity* | |
| *Predictors* | *Estimates* | *SE* | *Estimates* | *SE* |
| (Intercept) | 1106.49^***^ | 17.67 | 994.28^***^ | 20.04 |
| Duration | 0.52^***^ | 0.00 | 0.52^***^ | 0.00 |
| Number of phonemes | -5.50^***^ | 0.53 | -5.51^***^ | 0.53 |
| Familiarity | -53.45^***^ | 2.75 | -52.22^***^ | 2.77 |
| Prevalence | -47.83^***^ | 4.37 | -46.57^***^ | 4.38 |
| PND | 0.89^***^ | 0.04 | 0.88^***^ | 0.04 |
| **SUBTLEX-UK Zipf Value** | **-17.26^***^** | **1.31** |  |  |
| **Log_10_(CD_SUBTLEX-UK)** |  |  | **-20.01^***^** | **1.46** |
| **Random Effects** | |  |  |  |
| σ^2^ | 47419.83 |  | 47419.80 |  |
| τ_00_ | 4932.80 _word_ |  | 4921.29 _word_ |  |
|  | 6874.32 _subject_ |  | 6874.58 _subject_ |  |
| Marginal R^2^ | 0.120 |  | 0.120 |  |

*Note: ***, <.001; **,<.01; *, <.05*

**Tabe S 3** Linear mixed-effects model results for the effects of Zipf Value and Contextual Diversity from SUBTLEX-US on Response Time in the British Accent Auditory Lexical Decision Task (Goh et al., 2020). CD = Contextual Diversity; PND = Phonological Neighborhood Density; SE = Standard Error. Both PND and number of phonemes are based on Singaporean accent.

|  | *Response Time(linear mixed effect model)* | | | |
| --- | --- | --- | --- | --- |
|  | *Word frequency* | | *Contextual Diversity* | |
| *Predictors* | *Estimates* | *SE* | *Estimates* | *SE* |
| (Intercept) | 1118.60^***^ | 17.63 | 1044.46^***^ | 18.57 |
| Duration | 0.52^***^ | 0.00 | 0.52^***^ | 0.00 |
| Number of phonemes | -5.90^***^ | 0.54 | -6.00^***^ | 0.54 |
| Familiarity | -54.77^***^ | 2.74 | -53.33^***^ | 2.75 |
| Prevalence | -48.24^***^ | 4.37 | -45.77^***^ | 4.40 |
| PND | 0.89^***^ | 0.04 | 0.88^***^ | 0.04 |
| **SUBTLEX-US Zipf Value** | **-17.69**^***^ | **1.37** |  |  |
| **Log_10_(CD_SUBTLEX-US)** |  |  | **-20.94**^***^ | **1.53** |
| **Random Effects** | |  |  |  |
| σ^2^ | 47419.77 |  | 47419.74 |  |
| τ_00_ | 4937.72 _word_ |  | 4921.96 _word_ |  |
|  | 6873.01 _subject_ |  | 6873.63 _subject_ |  |
| Marginal R^2^ | 0.120 |  | 0.120 |  |

*Note: ***, <.001; **,<.01; *, <.05*

**Tabe S 4** Generalized linear mixed-effects model results for the effects of Zipf Value and Contextual Diversity from NSC on Accuracy in the British Accent Auditory Lexical Decision Task (Goh et al., 2020). CD = Contextual Diversity; PND = Phonological Neighborhood Density. Both PND and number of phonemes are based on Singaporean accent.

|  | *Accuracy(generalized linear mixed effect model)* | | | |
| --- | --- | --- | --- | --- |
|  | *Word frequency* | | *Contextual Diversity* | |
| *Predictors* | *Odds Ratios* | *SE* | *Odds Ratios* | *SE* |
| (Intercept) | 0.00^***^ | 0.00 | 0.01^***^ | 0.00 |
| Duration | 1.00^***^ | 0.00 | 1.00^***^ | 0.00 |
| Number of phonemes | 1.27^***^ | 0.01 | 1.28^***^ | 0.01 |
| Familiarity | 2.58^***^ | 0.10 | 2.50^***^ | 0.10 |
| Prevalence | 2.58^***^ | 0.16 | 2.56^***^ | 0.16 |
| PND | 0.99^***^ | 0.00 | 0.99^***^ | 0.00 |
| **NSC Zipf Value** | **1.33^***^** | **0.02** |  |  |
| **Log_10_(CD_NSC)** |  |  | **1.36^***^** | **0.02** |
| **Random Effects** | |  |  |  |
| σ^2^ | 3.29 |  | 3.29 |  |
| τ_00_ | 0.48 _subject_ |  | 0.48 _subject_ |  |
|  | 0.98 _word_ |  | 0.98 _word_ |  |
| Marginal R^2^ | 0.132 |  | 0.133 |  |

*Note: ***, <.001; **,<.01; *, <.05*

**Tabe S 5** Generalized linear mixed-effects model results for the effects of Zipf Value and Contextual Diversity from SUBTLEX-UK on Accuracy in the British Accent Auditory Lexical Decision Task (Goh et al., 2020). CD = Contextual Diversity; PND = Phonological Neighborhood Density. Both PND and number of phonemes are based on Singaporean accent.

|  | *Accuracy(generalized linear mixed effect model)* | | | |
| --- | --- | --- | --- | --- |
|  | *Word frequency* | | *Contextual Diversity* | |
| *Predictors* | *Odds Ratios* | *SE* | *Odds Ratios* | *SE* |
| (Intercept) | 0.00^***^ | 0.00 | 0.00^***^ | 0.00 |
| Duration | 1.00^***^ | 0.00 | 1.00^***^ | 0.00 |
| Number of phonemes | 1.28^***^ | 0.01 | 1.28^***^ | 0.01 |
| Familiarity | 2.82^***^ | 0.11 | 2.75^***^ | 0.11 |
| Prevalence | 2.35^***^ | 0.16 | 2.28^***^ | 0.15 |
| PND | 0.99^***^ | 0.00 | 0.99^***^ | 0.00 |
| **SUBTLEX-UK Zipf Value** | 1.37^***^ | 0.03 |  |  |
| **Log_10_(CD_SUBTLEX-UK)** |  |  | 1.44^***^ | 0.03 |
| **Random Effects** | |  |  |  |
| σ^2^ | 3.29 |  | 3.29 |  |
| τ_00_ | 0.48 _subject_ |  | 0.48 _subject_ |  |
|  | 1.00 _word_ |  | 0.99 _word_ |  |
| Marginal R^2^ | 0.130 |  | 0.131 |  |

*Note: ***, <.001; **,<.01; *, <.05*

**Tabe S 6** Generalized linear mixed-effects model results for the effects of Zipf Value and Contextual Diversity from SUBTLEX-US on Accuracy in the British Accent Auditory Lexical Decision Task (Goh et al., 2020). CD = Contextual Diversity; PND = Phonological Neighborhood Density. Both PND and number of phonemes are based on Singaporean accent.

|  | *Accuracy(generalized linear mixed effect model)* | | | |
| --- | --- | --- | --- | --- |
|  | *Word frequency* | | *Contextual Diversity* | |
| *Predictors* | *Odds Ratios* | *SE* | *Odds Ratios* | *SE* |
| (Intercept) | 0.00^***^ | 0.00 | 0.00^***^ | 0.00 |
| Duration | 1.00^***^ | 0.00 | 1.00^***^ | 0.00 |
| Number of phonemes | 1.28^***^ | 0.01 | 1.29^***^ | 0.01 |
| Familiarity | 2.90^***^ | 0.11 | 2.83^***^ | 0.11 |
| Prevalence | 2.40^***^ | 0.16 | 2.28^***^ | 0.15 |
| PND | 0.99^***^ | 0.00 | 0.99^***^ | 0.00 |
| **SUBTLEX-US Zipf Value** | **1.35^***^** | **0.03** |  |  |
| **Log_10_(CD_SUBTLEX-US)** |  |  | **1.44^***^** | **0.04** |
| **Random Effects** | |  |  |  |
| σ^2^ | 3.29 |  | 3.29 |  |
| τ_00_ | 0.48 _subject_ |  | 0.48 _subject_ |  |
|  | 1.00 _word_ |  | 0.99 _word_ |  |
| Marginal R^2^ | 0.129 |  | 0.130 |  |

*Note: ***, <.001; **,<.01; *, <.05*

**Tabe S 7** Linear mixed-effects model results for the effects of Zipf Value and Contextual Diversity from NSC on Response Time in the American Accent Auditory Lexical Decision Task (Goh et al., 2020). CD = Contextual Diversity; PND = Phonological Neighborhood Density; SE = Standard Error. Both PND and number of phonemes are based on Singaporean accent.

|  | *Response Time(linear mixed effect model)* | | | |
| --- | --- | --- | --- | --- |
|  | *Word frequency* | | *Contextual Diversity* | |
| *Predictors* | *Estimates* | *SE* | *Estimates* | *SE* |
| (Intercept) | 1178.30^***^ | 17.72 | 1082.15^***^ | 20.30 |
| Duration | 0.41^***^ | 0.00 | 0.41^***^ | 0.00 |
| Number of phonemes | 0.33 | 0.51 | 0.31 | 0.50 |
| Familiarity | -50.46^***^ | 2.71 | -48.98^***^ | 2.73 |
| Prevalence | -60.91^***^ | 4.06 | -60.64^***^ | 4.05 |
| PND | 0.80^***^ | 0.04 | 0.79^***^ | 0.04 |
| **NSC Zipf Value** | **-13.85^***^** | **1.02** |  |  |
| **Log_10_(CD_NSC)** |  |  | **-14.82**^***^ | **1.07** |
| **Random Effects** | |  |  |  |
| σ^2^ | 49007.73 |  | 49007.76 |  |
| τ_00_ | 4632.02 _word_ |  | 4626.46 _word_ |  |
|  | 9550.31 _subject_ |  | 9551.67 _subject_ |  |
| Marginal R^2^ | 0.071 |  | 0.071 |  |

*Note: ***, <.001; **,<.01; *, <.05*

**Tabe S 8** Linear mixed-effects model results for the effects of Zipf Value and Contextual Diversity from SUBTLEX-UK on Response Time in the American Accent Auditory Lexical Decision Task (Goh et al., 2020). CD = Contextual Diversity; PND = Phonological Neighborhood Density; SE = Standard Error. Both PND and number of phonemes are based on Singaporean accent.

|  | *Response Time(linear mixed effect model)* | | | |
| --- | --- | --- | --- | --- |
|  | *Word frequency* | | *Contextual Diversity* | |
| *Predictors* | *Estimates* | *SE* | *Estimates* | *SE* |
| (Intercept) | 1210.17**^***^** | 17.41 | 1106.72**^***^** | 19.64 |
| Duration | 0.41**^***^** | 0.00 | 0.41**^***^** | 0.00 |
| Number of phonemes | 0.10 | 0.51 | 0.12 | 0.51 |
| Familiarity | -54.64**^***^** | 2.64 | -53.57**^***^** | 2.65 |
| Prevalence | -55.45**^***^** | 4.21 | -54.48**^***^** | 4.21 |
| PND | 0.81**^***^** | 0.04 | 0.81**^***^** | 0.04 |
| **SUBTLEX-UK Zipf Value** | **-16.11^***^** | **1.27** |  |  |
| **Log_10_(CD_SUBTLEX-UK)** |  |  | **-18.51^***^** | **1.40** |
| **Random Effects** | |  |  |  |
| σ^2^ | 49007.52 |  | 49007.60 |  |
| τ_00_ | 4647.41 _word_ |  | 4638.95 _word_ |  |
|  | 9549.40 _subject_ |  | 9549.36 _subject_ |  |
| Marginal R^2^ | 0.071 |  | 0.071 |  |

*Note: ***, <.001; **,<.01; *, <.05*

**Tabe S 9** Linear mixed-effects model results for the effects of Zipf Value and Contextual Diversity from SUBTLEX-US on Response Time in the American Accent Auditory Lexical Decision Task (Goh et al., 2020). CD = Contextual Diversity; PND = Phonological Neighborhood Density; SE = Standard Error. Both PND and number of phonemes are based on Singaporean accent.

|  | *Response Time(linear mixed effect model)* | | | |
| --- | --- | --- | --- | --- |
|  | *Word frequency* | | *Contextual Diversity* | |
| *Predictors* | *Estimates* | *SE* | *Estimates* | *SE* |
| (Intercept) | 1220.72^***^ | 17.34 | 1144.30^***^ | 18.22 |
| Duration | 0.41^***^ | 0.00 | 0.41^***^ | 0.00 |
| Number of phonemes | -0.45 | 0.52 | -0.54 | 0.52 |
| Familiarity | -55.28^***^ | 2.62 | -53.82^***^ | 2.63 |
| Prevalence | -53.74^***^ | 4.20 | -51.28^***^ | 4.22 |
| PND | 0.83^***^ | 0.04 | 0.82^***^ | 0.04 |
| **SUBTLEX-US Zipf Value** | **-18.36^***^** | **1.32** |  |  |
| **Log_10_(CD_SUBTLEX-US)** |  |  | **-21.64**^***^ | **1.47** |
| **Random Effects** | |  |  |  |
| σ^2^ | 49007.49 |  | 49007.60 |  |
| τ_00_ | 4626.49 _word_ |  | 4609.96 _word_ |  |
|  | 9549.74 _subject_ |  | 9548.82 _subject_ |  |
| Marginal R^2^ | 0.071 |  | 0.071 |  |

*Note: ***, <.001; **,<.01; *, <.05*

**Tabe S 10** Generalized linear mixed-effects model results for the effects of Zipf Value and Contextual Diversity from NSC on Accuracy in the American Accent Auditory Lexical Decision Task (Goh et al., 2020). CD = Contextual Diversity; PND = Phonological Neighborhood Density. Both PND and number of phonemes are based on Singaporean accent.

|  | *Accuracy(generalized linear mixed effect model)* | | | |
| --- | --- | --- | --- | --- |
|  | *Word frequency* | | *Contextual Diversity* | |
| *Predictors* | *Odds Ratios* | *SE* | *Odds Ratios* | *SE* |
| (Intercept) | 0.00**^***^** | 0.00 | 0.00**^***^** | 0.00 |
| Duration | 1.00**^**^** | 0.00 | 1.00**^**^** | 0.00 |
| Number of phonemes | 1.23**^***^** | 0.01 | 1.23**^***^** | 0.01 |
| Familiarity | 2.65**^***^** | 0.11 | 2.57**^***^** | 0.11 |
| Prevalence | 3.07**^***^** | 0.21 | 3.03**^***^** | 0.20 |
| PND | 0.99**^***^** | 0.00 | 0.99**^***^** | 0.00 |
| **NSC Zipf Value** | 1.27**^***^** | 0.02 |  |  |
| **Log_10_(CD_NSC)** |  |  | **1.30^***^** | **0.02** |
| **Random Effects** | |  |  |  |
| σ^2^ | 3.29 |  | 3.29 |  |
| τ_00_ | 0.47 _subject_ |  | 0.47 _subject_ |  |
|  | 1.12 _word_ |  | 1.11 _word_ |  |
| Marginal R^2^ | 0.124 |  | 0.125 |  |

*Note: ***, <.001; **,<.01; *, <.05*

**Tabe S 11** Generalized linear mixed-effects model results for the effects of Zipf Value and Contextual Diversity from SUBTLEX-UK on Accuracy in the American Accent Auditory Lexical Decision Task (Goh et al., 2020). CD = Contextual Diversity; PND = Phonological Neighborhood Density. Both PND and number of phonemes are based on Singaporean accent.

|  | *Accuracy(generalized linear mixed effect model)* | | | |
| --- | --- | --- | --- | --- |
|  | *Word frequency* | | *Contextual Diversity* | |
| *Predictors* | *Odds Ratios* | *SE* | *Odds Ratios* | *SE* |
| (Intercept) | 0.00^***^ | 0.00 | 0.00^***^ | 0.00 |
| Duration | 1.00^*^ | 0.00 | 1.00^*^ | 0.00 |
| Number of phonemes | 1.23^***^ | 0.01 | 1.23^***^ | 0.01 |
| Familiarity | 2.86^***^ | 0.12 | 2.80^***^ | 0.11 |
| Prevalence | 2.84^***^ | 0.20 | 2.75^***^ | 0.19 |
| PND | 0.99^***^ | 0.00 | 0.99^***^ | 0.00 |
| **SUBTLEX-UK Zipf Value** | **1.30^***^** | **0.03** |  |  |
| **Log_10_(CD_SUBTLEX-UK)** |  |  | **1.37^***^** | **0.03** |
| **Random Effects** | |  |  |  |
| σ^2^ | 3.29 |  | 3.29 |  |
| τ_00_ | 0.47 _subject_ |  | 0.47 _subject_ |  |
|  | 1.13 _word_ |  | 1.12 _word_ |  |
| Marginal R^2^ | 0.123 |  | 0.124 |  |

*Note: ***, <.001; **,<.01; *, <.05*

**Tabe S 12** Generalized linear mixed-effects model results for the effects of Zipf Value and Contextual Diversity from SUBTLEX-US on Accuracy in the American Accent Auditory Lexical Decision Task (Goh et al., 2020). CD = Contextual Diversity; PND = Phonological Neighborhood Density. Both PND and number of phonemes are based on Singaporean accent.

|  | *Accuracy(generalized linear mixed effect model)* | | | |
| --- | --- | --- | --- | --- |
|  | *Word frequency* | | *Contextual Diversity* | |
| *Predictors* | *Odds Ratios* | *SE* | *Odds Ratios* | *SE* |
| (Intercept) | 0.00**^***^** | 0.00 | 0.00**^***^** | 0.00 |
| Duration | 1.00**^*^** | 0.00 | 1.00**^*^** | 0.00 |
| Number of phonemes | 1.24**^***^** | 0.01 | 1.25**^***^** | 0.01 |
| Familiarity | 2.89**^***^** | 0.12 | 2.82**^***^** | 0.11 |
| Prevalence | 2.75**^***^** | 0.19 | 2.61**^***^** | 0.18 |
| PND | 0.99**^***^** | 0.00 | 0.99**^***^** | 0.00 |
| **SUBTLEX-US Zipf Value** | **1.35^***^** | **0.03** |  |  |
| **Log_10_(CD_SUBTLEX-US)** |  |  | **1.44^***^** | **0.04** |
| **Random Effects** | |  |  |  |
| σ^2^ | 3.29 |  | 3.29 |  |
| τ_00_ | 0.47 _subject_ |  | 0.47 _subject_ |  |
|  | 1.12 _word_ |  | 1.11 _word_ |  |
| Marginal R^2^ | 0.124 |  | 0.125 |  |

*Note: ***, <.001; **,<.01; *, <.05*

## Model summaries with the SUBTLEX-US and SUBTLEX-UK for Singaporean, British and American accent in the manuscript.

**Tabe S 13** Linear mixed-effects model results for the effects of Zipf Value and Contextual Diversity from SUBTLEX-UK on Response Time in the Singaporean Accent Auditory Lexical Decision Task (Goh et al., 2020). CD = Contextual Diversity; PND = Phonological Neighborhood Density; SE = Standard Error. Both PND and number of phonemes are based on Singaporean accent.

|  | *Response Time(linear mixed effect model)* | | | |
| --- | --- | --- | --- | --- |
|  | *Word frequency* | | *Contextual Diversity* | |
| *Predictors* | *Estimates* | *SE* | *Estimates* | *SE* |
| (Intercept) | 1136.87^***^ | 16.96 | 1042.49^***^ | 19.15 |
| Duration | 0.55^***^ | 0.00 | 0.55^***^ | 0.00 |
| Number of phonemes | -7.96^***^ | 0.51 | -7.94^***^ | 0.50 |
| Familiarity | -61.20^***^ | 2.59 | -60.25^***^ | 2.60 |
| Prevalence | -40.06^***^ | 4.09 | -39.27^***^ | 4.09 |
| PND | 0.83^***^ | 0.04 | 0.83^***^ | 0.04 |
| **SUBTLEX-UK Zipf Value** | **-14.80^***^** | **1.23** |  |  |
| **Log_10_(CD_SUBTLEX-UK)** |  |  | **-16.90**^***^ | **1.36** |
| **Random Effects** | |  |  |  |
| σ^2^ | 47152.88 |  | 47152.87 |  |
| τ_00_ | 4164.87 _word_ |  | 4159.51 _word_ |  |
|  | 7048.02 _subject_ |  | 7047.39 _subject_ |  |
| Marginal R^2^ | 0.162 |  | 0.162 |  |

*Note: ***, <.001; **,<.01; *, <.05*

**Tabe S 14** Linear mixed-effects model results for the effects of Zipf Value and Contextual Diversity from SUBTLEX-US on Response Time in the Singaporean Accent Auditory Lexical Decision Task (Goh et al., 2020). CD = Contextual Diversity; PND = Phonological Neighborhood Density; SE = Standard Error. Both PND and number of phonemes are based on Singaporean accent.

|  | *Response Time(linear mixed effect model)* | | | |
| --- | --- | --- | --- | --- |
|  | *Word frequency* | | *Contextual Diversity* | |
| *Predictors* | *Estimates* | *SE* | *Estimates* | *SE* |
| (Intercept) | 1147.13^***^ | 16.91 | 1080.58^***^ | 17.78 |
| Duration | 0.55^***^ | 0.00 | 0.55^***^ | 0.00 |
| Number of phonemes | -8.34^***^ | 0.51 | -8.45^***^ | 0.51 |
| Familiarity | -62.18^***^ | 2.57 | -60.80^***^ | 2.58 |
| Prevalence | -39.85^***^ | 4.08 | -37.41^***^ | 4.11 |
| PND | 0.84^***^ | 0.04 | 0.84^***^ | 0.04 |
| **SUBTLEX-US Zipf Value** | **-15.66**^***^ | **1.28** |  |  |
| **Log_10_(CD_SUBTLEX-US)** |  |  | **-18.78**^***^ | **1.42** |
| **Random Effects** | |  |  |  |
| σ^2^ | 47152.93 |  | 47152.97 |  |
| τ_00_ | 4162.25 _word_ |  | 4146.68 _word_ |  |
|  | 7046.97 _subject_ |  | 7046.53 _subject_ |  |
| Marginal R^2^ | 0.161 |  | 0.162 |  |

*Note: ***, <.001; **,<.01; *, <.05*

**Tabe S 15** Generalized linear mixed-effects model results for the effects of Zipf Value and Contextual Diversity from SUBTLEX-UK on Accuracy in the Singaporean Accent Auditory Lexical Decision Task (Goh et al., 2020). CD = Contextual Diversity; PND = Phonological Neighborhood Density. Both PND and number of phonemes are based on Singaporean accent.

|  | *Accuracy(generalized linear mixed effect model)* | | | |
| --- | --- | --- | --- | --- |
|  | *Word frequency* | | *Contextual Diversity* | |
| *Predictors* | *Odds Ratios* | *SE* | *Odds Ratios* | *SE* |
| (Intercept) | 0.00^***^ | 0.00 | 0.00^***^ | 0.00 |
| Duration | 1.00^***^ | 0.00 | 1.00^***^ | 0.00 |
| Number of phonemes | 1.25^***^ | 0.01 | 1.25^***^ | 0.01 |
| Familiarity | 3.03^***^ | 0.11 | 2.98^***^ | 0.11 |
| Prevalence | 2.03^***^ | 0.13 | 1.98^***^ | 0.13 |
| PND | 0.99^***^ | 0.00 | 0.99^***^ | 0.00 |
| **SUBTLEX-UK Zipf Value** | 1.25^***^ | 0.03 |  |  |
| **Log_10_(CD_SUBTLEX-UK)** |  |  | 1.31 | 0.03 |
| **Random Effects** | |  |  |  |
| σ^2^ | 3.29 |  | 3.29 |  |
| τ_00_ | 0.39 _subject_ |  | 0.39 _subject_ |  |
|  | 0.91 _word_ |  | 0.91 _word_ |  |
| Marginal R^2^ | 0.119 |  | 0.119 |  |

*Note: ***, <.001; **,<.01; *, <.05*

**Tabe S 16** Generalized linear mixed-effects model results for the effects of Zipf Value and Contextual Diversity from SUBTLEX-US on Accuracy in the Singaporean Accent Auditory Lexical Decision Task (Goh et al., 2020). CD = Contextual Diversity; PND = Phonological Neighborhood Density. Both PND and number of phonemes are based on Singaporean accent.

|  | *Accuracy(generalized linear mixed effect model)* | | | |
| --- | --- | --- | --- | --- |
|  | *Word frequency* | | *Contextual Diversity* | |
| *Predictors* | *Odds Ratios* | *SE* | *Odds Ratios* | *SE* |
| (Intercept) | 0.00^***^ | 0.00 | 0.00^***^ | 0.00 |
| Duration | 1.00^***^ | 0.00 | 1.00^***^ | 0.00 |
| Number of phonemes | 1.25^***^ | 0.01 | 1.25^***^ | 0.01 |
| Familiarity | 3.09^***^ | 0.11 | 3.03^***^ | 0.11 |
| Prevalence | 2.06^***^ | 0.13 | 1.96^***^ | 0.13 |
| PND | 0.99^***^ | 0.00 | 0.99^***^ | 0.00 |
| **SUBTLEX-US Zipf Value** | **1.24^***^** | **0.03** |  |  |
| **Log_10_(CD_SUBTLEX-US)** |  |  | **1.31** | **0.03** |
| **Random Effects** | |  |  |  |
| σ^2^ | 3.29 |  | 3.29 |  |
| τ_00_ | 0.39 _subject_ |  | 0.39 _subject_ |  |
|  | 0.91 _word_ |  | 0.91 _word_ |  |
| Marginal R^2^ | 0.118 |  | 0.119 |  |

*Note: ***, <.001; **,<.01; *, <.05*

**Tabe S 17** Linear mixed-effects model results for the effects of Zipf Value and Contextual Diversity from SUBTLEX-UK on Response Time in the British Accent Auditory Lexical Decision Task (Goh et al., 2020). CD = Contextual Diversity; PND = Phonological Neighborhood Density; SE = Standard Error. Both PND and number of phonemes are based on British accent.

|  | *Response Time(linear mixed effect model)* | | | |
| --- | --- | --- | --- | --- |
|  | *Word frequency* | | *Contextual Diversity* | |
| *Predictors* | *Estimates* | *SE* | *Estimates* | *SE* |
| (Intercept) | 1067.06**^***^** | 16.25 | 945.12**^***^** | 18.53 |
| Duration | 0.52**^***^** | 0.00 | 0.52**^***^** | 0.00 |
| Number of phonemes | -3.35**^***^** | 0.54 | -3.35**^***^** | 0.53 |
| Familiarity | -49.84**^***^** | 2.52 | -48.57**^***^** | 2.53 |
| Prevalence | -46.70**^***^** | 4.21 | -45.37**^***^** | 4.22 |
| PND | 3.68**^***^** | 0.14 | 3.68**^***^** | 0.14 |
| **SUBTLEX-UK Zipf Value** | **-18.86^***^** | **1.27** |  |  |
| **Log_10_(CD_SUBTLEX-UK)** |  |  | **-21.76^***^** | **1.41** |
| **Random Effects** | |  |  |  |
| σ^2^ | 47663.22 |  | 47663.21 |  |
| τ_00_ | 4837.77 _word_ |  | 4824.84 _word_ |  |
|  | 6933.92 _subject_ |  | 6933.31 _subject_ |  |
| Marginal R^2^ | 0.124 |  | 0.162 |  |

*Note: ***, <.001; **,<.01; *, <.05*

**Tabe S 18** Linear mixed-effects model results for the effects of Zipf Value and Contextual Diversity from SUBTLEX-US on Response Time in the British Accent Auditory Lexical Decision Task (Goh et al., 2020). CD = Contextual Diversity; PND = Phonological Neighborhood Density; SE = Standard Error. Both PND and number of phonemes are based on British accent.

|  | *Response Time(linear mixed effect model)* | | | |
| --- | --- | --- | --- | --- |
|  | *Word frequency* | | *Contextual Diversity* | |
| *Predictors* | *Estimates* | *SE* | *Estimates* | *SE* |
| (Intercept) | 1080.81^***^ | 16.22 | 1000.76^***^ | 17.08 |
| Duration | 0.52^***^ | 0.00 | 0.52^***^ | 0.00 |
| Number of phonemes | -3.76^***^ | 0.54 | -3.86^***^ | 0.54 |
| Familiarity | -51.37^***^ | 2.50 | -49.92^***^ | 2.51 |
| Prevalence | -47.30^***^ | 4.21 | -44.61^***^ | 4.24 |
| PND | 3.70^***^ | 0.14 | 3.69^***^ | 0.14 |
| **SUBTLEX-US Zipf Value** | **-19.26^***^** | **1.34** |  |  |
| **Log_10_(CD_SUBTLEX-US)** |  |  | **-22.74^***^** | **1.48** |
| **Random Effects** | |  |  |  |
| σ^2^ | 47663.12 |  | 47663.12 |  |
| τ_00_ | 4846.20 _word_ |  | 4827.05 _word_ |  |
|  | 6932.39 _subject_ |  | 6932.78 _subject_ |  |
| Marginal R^2^ | 0.124 |  | 0.124 |  |

*Note: ***, <.001; **,<.01; *, <.05*

**Tabe S 19** Generalized linear mixed-effects model results for the effects of Zipf Value and Contextual Diversity from SUBTLEX-UK on Accuracy in the British Accent Auditory Lexical Decision Task (Goh et al., 2020). CD = Contextual Diversity; PND = Phonological Neighborhood Density. Both PND and number of phonemes are based on British accent.

|  | *Accuracy(generalized linear mixed effect model)* | | | |
| --- | --- | --- | --- | --- |
|  | *Word frequency* | | *Contextual Diversity* | |
| *Predictors* | *Odds Ratios* | *SE* | *Odds Ratios* | *SE* |
| (Intercept) | 0.00^***^ | 0.00 | 0.00^***^ | 0.00 |
| Duration | 1.00^***^ | 0.00 | 1.00^***^ | 0.00 |
| Number of phonemes | 1.26^***^ | 0.01 | 1.26^***^ | 0.01 |
| Familiarity | 2.73^***^ | 0.10 | 2.67^***^ | 0.10 |
| Prevalence | 2.32^***^ | 0.15 | 2.25^***^ | 0.15 |
| PND | 0.98^***^ | 0.00 | 0.98^***^ | 0.00 |
| **SUBTLEX-UK Zipf Value** | **1.38^***^** | **0.03** |  |  |
| **Log_10_(CD_SUBTLEX-UK)** |  |  | **1.46^***^** | **0.03** |
| **Random Effects** | |  |  |  |
| σ^2^ | 3.29 |  | 3.29 |  |
| τ_00_ | 0.47 _subject_ |  | 0.47 _subject_ |  |
|  | 1.02 _word_ |  | 1.01 _word_ |  |
| Marginal R^2^ | 0.132 |  | 0.133 |  |

*Note: ***, <.001; **,<.01; *, <.05*

**Tabe S 20** Generalized linear mixed-effects model results for the effects of Zipf Value and Contextual Diversity from SUBTLEX-US on Accuracy in the British Accent Auditory Lexical Decision Task (Goh et al., 2020). CD = Contextual Diversity; PND = Phonological Neighborhood Density. Both PND and number of phonemes are based on British accent.

|  | *Accuracy(generalized linear mixed effect model)* | | | |
| --- | --- | --- | --- | --- |
|  | *Word frequency* | | *Contextual Diversity* | |
| *Predictors* | *Odds Ratios* | *SE* | *Odds Ratios* | *SE* |
| (Intercept) | 0.00^***^ | 0.00 | 0.00^***^ | 0.00 |
| Duration | 1.00^***^ | 0.00 | 1.00^***^ | 0.00 |
| Number of phonemes | 1.27^***^ | 0.01 | 1.27^***^ | 0.01 |
| Familiarity | 2.81^***^ | 0.10 | 2.74^***^ | 0.10 |
| Prevalence | 2.36^***^ | 0.15 | 2.24^***^ | 0.15 |
| PND | 0.98^***^ | 0.00 | 0.98^***^ | 0.00 |
| **SUBTLEX-US Zipf Value** | **1.37^***^** | **0.03** |  |  |
| **Log_10_(CD_SUBTLEX-US)** |  |  | **1.47^***^** | **0.04** |
| **Random Effects** | |  |  |  |
| σ^2^ | 3.29 |  | 3.29 |  |
| τ_00_ | 0.47 _subject_ |  | 0.47 _subject_ |  |
|  | 1.02 _word_ |  | 1.01 _word_ |  |
| Marginal R^2^ | 0.131 |  | 0.132 |  |

*Note: ***, <.001; **,<.01; *, <.05*

**Tabe S 21** Linear mixed-effects model results for the effects of Zipf Value and Contextual Diversity from SUBTLEX-UK on Response Time in the American Accent Auditory Lexical Decision Task (Goh et al., 2020). CD = Contextual Diversity; PND = Phonological Neighborhood Density; SE = Standard Error. Both PND and number of phonemes are based on American accent.

|  | *Response Time(linear mixed effect model)* | | | |
| --- | --- | --- | --- | --- |
|  | *Word frequency* | | *Contextual Diversity* | |
| *Predictors* | *Estimates* | *SE* | *Estimates* | *SE* |
| (Intercept) | 1174.74^***^ | 16.22 | 1063.50^***^ | 18.38 |
| Duration | 0.41^***^ | 0.00 | 0.41^***^ | 0.00 |
| Number of phonemes | 1.99^***^ | 0.51 | 2.00^***^ | 0.51 |
| Familiarity | -51.35^***^ | 2.44 | -50.21^***^ | 2.45 |
| Prevalence | -53.98^***^ | 4.09 | -52.84^***^ | 4.09 |
| PND | 3.46^***^ | 0.15 | 3.46^***^ | 0.15 |
| **SUBTLEX-UK Zipf Value** | **-17.29^***^** | **1.24** |  |  |
| **Log_10_(CD_SUBTLEX-UK)** |  |  | **-19.89**^***^ | **1.37** |
| **Random Effects** | |  |  |  |
| σ^2^ | 49358.36 |  | 49358.46 |  |
| τ_00_ | 4661.11 _word_ |  | 4650.52 _word_ |  |
|  | 9637.44 _subject_ |  | 9637.66 _subject_ |  |
| Marginal R^2^ | 0.074 |  | 0.074 |  |

*Note: ***, <.001; **,<.01; *, <.05*

Tabe S 22 Linear mixed-effects model results for the effects of Zipf Value and Contextual Diversity from SUBTLEX-US on Response Time in the American Accent Auditory Lexical Decision Task (Goh et al., 2020). CD = Contextual Diversity; PND = Phonological Neighborhood Density; SE = Standard Error. Both PND and number of phonemes are based on American accent.

|  | *Response Time(linear mixed effect model)* | | | |
| --- | --- | --- | --- | --- |
|  | *Word frequency* | | *Contextual Diversity* | |
| *Predictors* | *Estimates* | *SE* | *Estimates* | *SE* |
| (Intercept) | 1187.14^***^ | 16.16 | 1106.42^***^ | 16.96 |
| Duration | 0.41^***^ | 0.00 | 0.41^***^ | 0.00 |
| Number of phonemes | 1.49^**^ | 0.52 | 1.37^**^ | 0.52 |
| Familiarity | -52.29^***^ | 2.42 | -50.81^***^ | 2.42 |
| Prevalence | -52.54^***^ | 4.08 | -49.73^***^ | 4.10 |
| PND | 3.51^***^ | 0.15 | 3.51^***^ | 0.15 |
| **SUBTLEX-US Zipf Value** | **-19.39**^***^ | **1.29** |  |  |
| **Log_10_(CD_SUBTLEX-US)** |  |  | **-23.00**^***^ | **1.44** |
| **Random Effects** | |  |  |  |
| σ^2^ | 49358.32 |  | 49358.44 |  |
| τ_00_ | 4642.52 _word_ |  | 4621.09 _word_ |  |
|  | 9636.99 _subject_ |  | 9636.96 _subject_ |  |
| Marginal R^2^ | 0.074 |  | 0.075 |  |

*Note: ***, <.001; **,<.01; *, <.05*

**Tabe S 23** Generalized linear mixed-effects model results for the effects of Zipf Value and Contextual Diversity from SUBTLEX-UK on Accuracy in the American Accent Auditory Lexical Decision Task (Goh et al., 2020). CD = Contextual Diversity; PND = Phonological Neighborhood Density. Both PND and number of phonemes are based on American accent.

|  | *Accuracy(generalized linear mixed effect model)* | | | |
| --- | --- | --- | --- | --- |
|  | *Word frequency* | | *Contextual Diversity* | |
| *Predictors* | *Odds Ratios* | *SE* | *Odds Ratios* | *SE* |
| (Intercept) | 0.00^***^ | 0.00 | 0.00^***^ | 0.00 |
| Duration | 1.00^*^ | 0.00 | 1.00^*^ | 0.00 |
| Number of phonemes | 1.23^***^ | 0.01 | 1.23^***^ | 0.01 |
| Familiarity | 2.79^***^ | 0.10 | 2.73^***^ | 0.10 |
| Prevalence | 2.68^***^ | 0.18 | 2.59^***^ | 0.18 |
| PND | 0.98^***^ | 0.00 | 0.98^***^ | 0.00 |
| **SUBTLEX-UK Zipf Value** | **1.32^***^** | **0.03** |  |  |
| **Log_10_(CD_SUBTLEX-UK)** |  |  | **1.39^***^** | **0.03** |
| **Random Effects** | |  |  |  |
| σ^2^ | 3.29 |  | 3.29 |  |
| τ_00_ | 0.48 _subject_ |  | 0.48 _subject_ |  |
|  | 1.14 _word_ |  | 1.13 _word_ |  |
| Marginal R^2^ | 0.125 |  | 0.126 |  |

*Note: ***, <.001; **,<.01; *, <.05*

**Tabe S 24** Generalized linear mixed-effects model results for the effects of Zipf Value and Contextual Diversity from SUBTLEX-US on Accuracy in the British Accent Auditory Lexical Decision Task (Goh et al., 2020). CD = Contextual Diversity; PND = Phonological Neighborhood Density. Both PND and number of phonemes are based on American accent.

|  | *Accuracy(generalized linear mixed effect model)* | | | |
| --- | --- | --- | --- | --- |
|  | *Word frequency* | | *Contextual Diversity* | |
| *Predictors* | *Odds Ratios* | *SE* | *Odds Ratios* | *SE* |
| (Intercept) | 0.00^***^ | 0.00 | 0.00^***^ | 0.00 |
| Duration | 1.00^*^ | 0.00 | 1.00^*^ | 0.00 |
| Number of phonemes | 1.24^***^ | 0.01 | 1.24^***^ | 0.01 |
| Familiarity | 2.83^***^ | 0.10 | 2.76^***^ | 0.10 |
| Prevalence | 2.60^***^ | 0.18 | 2.46^***^ | 0.17 |
| PND | 0.98^***^ | 0.00 | 0.98^***^ | 0.00 |
| **SUBTLEX-US Zipf Value** | **1.37^***^** | **0.03** |  |  |
| **Log_10_(CD_SUBTLEX-US)** |  |  | **1.46^***^** | **0.04** |
| **Random Effects** | |  |  |  |
| σ^2^ | 3.29 |  | 3.29 |  |
| τ_00_ | 0.48 _subject_ |  | 0.48 _subject_ |  |
|  | 1.13 _word_ |  | 1.12 _word_ |  |
| Marginal R^2^ | 0.126 |  | 0.128 |  |

*Note: ***, <.001; **,<.01; *, <.05*
